# Neutrophil Extracellular Traps: From Mechanistic Insights to Therapeutic Targeting

**DOI:** 10.1002/mco2.71101

**Published:** 2026-11-09

**Authors:** Hongjie Fan, Yurui Tong, Bufu Tang, Jihong Hu, Xuefeng Kan, Bin Liang

**Affiliations:** ^1^ Department of Radiology Union Hospital Tongji Medical College Huazhong University of Science and Technology Wuhan China; ^2^ Hubei Provincial Clinical Research Center For Precision Radiology & Interventional Medicine Wuhan China; ^3^ Hubei Provincial Key Laboratory of Molecular Imaging Wuhan China; ^4^ The Fourth School of Clinical Medicine China Medical University Shenyang China; ^5^ Department of Radiation Oncology Zhongshan Hospital Fudan University Shanghai China; ^6^ Department of Minimally Invasive Intervention The First Affiliated Hospital of Kunming Medical University Kunming China

**Keywords:** antitumor immunity, immunotherapy, multimodal signal processors, neutrophil extracellular traps, tumor microenvironment

## Abstract

Neutrophils represent the most abundant leukocytes in circulation and serve to protect the host from infection. Paradoxically, they also contribute to cancer by releasing extracellular traps known as neutrophil extracellular traps (NETs). These structures exert opposing effects on tumors, either promoting progression or suppressing growth, depending on the specific context. However, a comprehensive framework reconciling these dual roles is lacking. NETs can be reconceptualised as multimodal signal processors within the tumor microenvironment, with a primary focus on cancer while acknowledging relevance to certain inflammatory and autoimmune conditions. NETs function through three interconnected modalities: (1) biomechanical scaffolds that remodel tissue architecture via integrin–focal adhesion kinase (FAK) mechanotransduction; (2) immunometabolic hubs that drive glycolytic reprogramming and immune suppression; and (3) signal‐integration platforms that concentrate oncogenic cues for synergistic pathway activation. This systems‐level perspective, orchestrated by nodes such as hypoxia‐inducible factor 1‐alpha and integrin‐FAK, elucidates how microenvironmental inputs dictate NET phenotypic polarity. Furthermore, therapeutic implications are addressed, including biomarker‐guided stratification, optimal intervention timing during the perioperative period, dormancy phase, immunotherapy combinations, and strategies to disrupt modal crosstalk while preserving essential host defence. Future priorities include resolving NET heterogeneity and developing localised delivery systems. This roadmap enhances precision targeting of NETs in oncology and other fields.

## Graphical abstract

Neutrophil extracellular traps act as multimodal signal processors in cancer via three mechanisms: biomechanical scaffolds activating integrin–FAK signaling and excluding immune cells; immunometabolic hubs driving glycolysis and immunosuppression; and signal‐integration platforms synergistically activating oncogenic pathways. Therapeutic strategies targeting these modalities—DNase, metabolic inhibitors, FAK blockers, and cytokine antagonists—enable precision NET‐directed interventions in oncology.

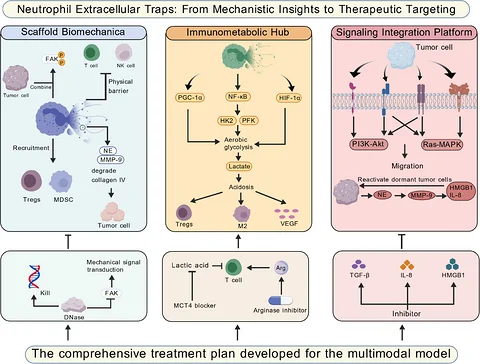

## 1 Introduction

Neutrophils, the most abundant circulating leukocytes [1], constitute the first line of cellular defense against invading pathogens [2]. Upon microbial challenge, neutrophils deploy various effector mechanisms—phagocytosis, degranulation, and reactive oxygen species (ROS) release—to contain and eliminate infectious threats [3, 4]. A seminal discovery expanded this paradigm with the identification of neutrophil extracellular traps (NETs): web‐like chromatin fibers decorated with histones and granule‐derived proteins that ensnare and neutralize bacteria, fungi, and viruses [5, 6]. NETs are released through a regulated process known as NETosis, which occurs via two principal pathways: the classical lytic form that culminates in plasma membrane rupture and neutrophil death [7, 8], and the nonlytic (vital) pathway, enabling rapid DNA extrusion while preserving cell viability [9, 10]. Over the past two decades, research has established that NETs serve roles far beyond antimicrobial defense, emerging as critical mediators in chronic inflammation, autoimmune diseases, and notably, cancer [11, 12, 13].

In oncology, NETs exhibit a context‐dependent duality: under certain conditions, they exert direct cytotoxic effects against tumor cells and contribute to immune surveillance; however, substantial evidence indicates that NETs predominantly promote tumor progression and metastasis by facilitating immune evasion, angiogenesis, thrombosis, and premetastatic niche formation [14, 15]. While numerous studies have catalogued these diverse functions, the field largely lacks a unifying conceptual framework that explains how NETs produce context‐dependent outcomes and what determinants govern their functional polarity. These questions directly influence therapeutic strategies, as effective targeting necessitates knowledge of when to inhibit NETs and when to preserve their functions.

The mechanistic principles underlying NET biology extend beyond cancer. In autoimmune joint disease, NETs act as physical scaffolds exacerbating inflammation and cartilage destruction; in cystic fibrosis, they create hypoxic, lactate‐rich microenvironments driving airway damage; and in systemic lupus erythematosus (SLE), they function as antigen‐presenting platforms that propagate autoimmunity [16]. Although this review primarily focuses on cancer—justified by the recent surge of mechanistic and translational data—the conceptual framework developed here has broader applicability across inflammatory, thrombotic, and autoimmune conditions.

To bridge the gap between descriptive cataloguing and mechanistic understanding, NETs are reconceptualised as multimodal signal processors within the tumor microenvironment (TME). This conceptual model posits that NETs operate through three interconnected and mutually reinforcing modalities: (1) biomechanical scaffolds that remodel tissue architecture and activate mechanotransduction via integrin–focal adhesion kinase (FAK) pathways; (2) immunometabolic hubs that drive glycolytic reprogramming, lactate accumulation, and amino acid depletion, linking metabolic alterations to immune suppression; and (3) signal‐integration platforms that spatially concentrate diverse oncogenic cues—cytokines, growth factors, proteases, and damage‐associated molecules—to achieve synergistic pathway activation (Figure 1). These modalities are dynamically interconnected through core interaction nodes—most notably hypoxia‐inducible factor 1‐alpha (HIF‐1α) and the integrin–FAK axis—which convert microenvironmental inputs into context‐dependent outputs: protumorigenic when chronic inflammation and hypoxia activate all three modalities, or antitumorigenic under acute interferon (IFN)‐γ‐rich conditions. This systems‐level perspective elucidates the dual role of NETs as an emergent property of the signal‐processing network and reveals therapeutic vulnerabilities, indicating that effective interventions require disrupting the interconnections that maintain the protumorigenic network state.

**FIGURE 1:**
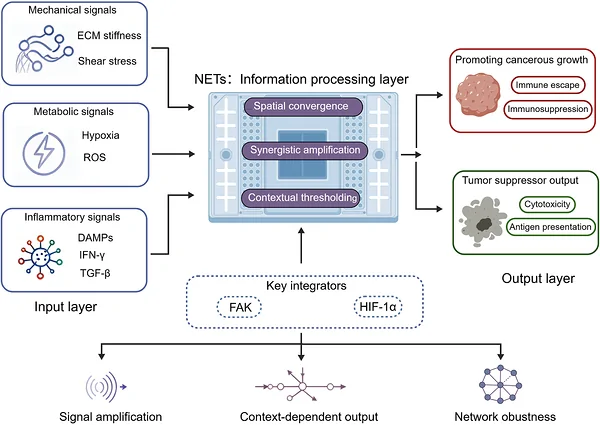
Conceptual framework of NETs as multimodal signal processors. This schematic depicts neutrophil extracellular traps as an integrated signal‐processing system organised into distinct functional layers. The left side displays the input layer, which consists of mechanical signals including ECM stiffness and shear stress, metabolic signals including hypoxia and reactive oxygen species, and inflammatory signals including damage‐associated molecular patterns, IFN‐γ, and TGF‐β. These diverse inputs converge upon the central information processing layer, which is characterised by three core operational features including spatial convergence, synergistic amplification, and contextual thresholding. Below this processing core, key integrators including FAK and HIF‐1α, serve as critical hubs for signal transduction and modality cross‐talk. The right side illustrates the output layer, which branches into two opposing functional states. The protumorigenic state promotes cancerous growth through immune escape and immunosuppression, whereas the antitumorigenic state mediates tumor suppressor output through cytotoxicity and antigen presentation. The bottom section further highlights the emergent systems‐level properties of this network, including signal amplification, context‐dependent output, and network robustness. This integrative framework explains the intrinsic duality of NETs and underscores the importance of targeting network‐level interactions rather than individual molecules for effective therapeutic intervention.

Subsequent sections review NET components and formation mechanisms, highlighting NETosis as a gatekeeper for signal integration. The multimodal framework is elaborated, detailing each modality and their interconnections through core nodes. Building on these mechanistic insights, tumor‐suppressive NET contexts and therapeutic implications, including biomarker‐guided stratification, optimal intervention timing, and rational combination strategies with immunotherapy, are explored. A critical examination of preclinical and clinical translation discusses unresolved controversies and negative data, explaining how this review advances the field. A forward‐looking perspective on key questions for future research concludes the review, emphasising the need for mechanistic clarity, improved biomarkers, localised delivery strategies, and cross‐disciplinary collaboration to translate NET‐targeted approaches into clinical practice.

## 2 Components of NETs

NETs contain numerous antibacterial elements, with proteases receiving the most attention. The DNA backbones extruded by neutrophils anchor these enzymes, enabling them to execute cytotoxic functions on target cells and proteolytic functions on extracellular matrix (ECM) components [17, 18]. Alongside proteases, NETs are enriched with matrix metalloproteinase 9 (MMP‐9), neutrophil elastase (NE), cathepsin G (CG), chemokines, antimicrobial peptides, and histones [19, 20, 21]. These molecules are intricately involved in endothelial injury, ECM regulation, vascular adhesion, and angiogenesis [22, 23]. Notably, NE, MMP‐9, CG, histones, and DNA are particularly relevant to cancer cell proliferation and tumor progression [20, 24].

NE, a serine protease found in neutrophil azurophilic granules [25], is the most abundant nonhistone protein of NETs and serves as one of the most potent bactericidal proteins released by neutrophils [26]. Beyond its antimicrobial action, NE acts as a critical mediator of inflammation, promoting microbial killing and inflammatory signaling [27, 28]. Additionally, NE contributes to metastasis and plays a role in TME remodeling within NET biology [29]. Mechanistically, NE directs tumor cell activation, cleaves tumor cell membrane ligands, stimulates angiogenesis, and interacts with other neutrophil‐derived mediators in the TME [30, 31]. It also cleaves protein recombinant CD95, producing proteolytic fragments containing the death domain that specifically bind histone H1 in malignant cells, leading to cytotoxicity [32]. At the cellular level, NE induces chromatin decondensation during NETosis, involving migration into the nucleus, utilization of histones, and disintegration of actin filaments [33, 34]. Myeloperoxidase (MPO) works in concert with NE to accelerate chromatin decondensation, a process that is partly independent of MPO's enzymatic activity [35]. Interestingly, the biological effects of NE are concentration dependent; low levels of NE can activate proinflammatory cytokines to their mature forms, amplifying proinflammatory effects [36]. NE also cleaves ligand‐binding sites of Toll‐like receptors (TLRs) on the cell surface and other bacterial recognition receptors, negatively regulating excessive activation of neutrophils to prevent overactivation, which paradoxically may modulate inflammatory responses and indirectly influence tumor cell behavior [37]. When NE concentrations exceed a certain threshold and endogenous inhibitors can no longer buffer them, uncontrolled hydrolytic activity ensues. High levels of NE directly degrade the ECM, causing damage to endothelial and epithelial cells and potentially resulting in extensive tissue destruction [23].

The MMP family of proteases plays a critical role in tissue development, wound healing, organ morphogenesis, and angiogenesis [38]. Among them, MMP‐9 is consistently upregulated in multiple malignancies and is strongly associated with tumor growth and invasion [39]. Released from neutrophils during degranulation, MMP‐9 enhances tumor progression by degrading ECM structures [40], suppressing apoptosis, and facilitating angiogenesis and metastatic dissemination [41, 42]. Additionally, NET‐associated MMP‐9 contributes to vascular dysfunction by activating pro‐MMP‐2 in endothelial cells (ECs), inducing endothelial injury and cell death [43]. MMP‐8 degrades the ECM, promotes tissue damage, and can process and activate various inflammatory‐related molecules, thereby regulating the inflammatory response. MMP‐25 primarily functions to activate other MMPs and can induce apoptosis in specific contexts.

CG, stored in azurophilic granules, participates in microbial killing both within phagolysosomes and in the extracellular space. When bound to NET‐DNA, CG resists inhibition by endogenous protease inhibitors, thereby sustaining its proteolytic activity [44]. This capability allows CG to degrade ECM proteins, including collagen, elastin, and laminin. In tumor settings, neutrophil‐derived DNA enhances the hydrolytic activity of CG by attenuating local protease inhibitors, amplifying CG‐mediated proteolysis [45, 46]. Additionally, CG can activate platelets, increasing its deposition on the tumor cell surface [47, 48].

Histones, another abundant component of NETs, exhibit direct cytotoxicity toward ECs [49]. At toxic concentrations, histones induce cell death and elicit strong proinflammatory signaling, surpassing the effects of DNA alone. Furthermore, NET‐derived DNA can function as a chemotactic factor, promoting tumor cell migration and facilitating metastatic spread [50]. Collectively, these NET‐associated components cooperate to remodel the extracellular environment, regulate vascular function, and shape cellular interactions, thereby fostering conditions conducive to tumor progression. Proteinaceous particles released during NETosis associate with the DNA backbone; for example, calprotectin limits microbial growth by sequestering zinc, while lactoferrin chelates iron and disrupts bacterial biofilms.

NETs exhibit significant compositional heterogeneity, meaning the components can vary across different tumor types. Research in this area, although not exhaustive, has been conducted in gastric cancer (GC) and ovarian cancer. In GC, expression levels of complement component 5a receptor 1 (C5AR1) significantly increase and are enriched in C5AR1‐related signaling pathways. Knocking out the C5AR1 gene has been shown to significantly inhibit the growth of GC cells, although the precise biological role of C5AR1 in GC remains undetermined [51]. These NETs may promote tumor progression by recruiting and activating other cell types within the TME. Additionally, peptidylprolyl isomerase F (PPIF) may emerge as a potential target for colorectal cancer treatment. In ovarian cancer, NETs are abundant in interleukin‐8 (IL‐8), granulocyte colony‐stimulating factor, and C–X–C motif chemokine ligands 1/2 (CXCL1/2), which promote peritoneal implantation [52].

Critically, this compositional heterogeneity translates into functional differences within the multimodal framework. NETs enriched in proteases such as NE and MMP‐9 predominantly engage the biomechanical scaffold modality by degrading ECM components, remodeling tissue architecture, and creating migration tracks that facilitate tumor cell invasion. Conversely, NETs rich in chemokines and inflammatory mediators primarily operate through the signal‐integration platform modality, concentrating diverse oncogenic cues to achieve synergistic receptor activation and sustained downstream signaling. NETs characterized by high levels of Arginase‐1 (Arg‐1) or lactate‐generating enzymes preferentially enhance the immunometabolic hub modality, depleting essential amino acids and creating an acidic, immunosuppressive microenvironment that impairs T cell function and promotes regulatory cell polarization. Thus, the functional output of NETs in any given tumor is critically dependent on the specific molecular composition of the formed NETs rather than merely their presence or absence.

This heterogeneity extends to the transcriptional level, with recent studies demonstrating that expression profiles of NET‐related genes vary substantially across tumor types. In GC, characteristic genes including C5AR1, CSF1R, CSF2RB, CYBB, HCK, ITGB2, LILRB2, MNDA, MPEG1, PLEK, SRGN, and STAB1 show aberrant expression [51]. In lung adenocarcinoma, a distinct set of genes such as CAT, CTSG, TLR2, and ENO1 predominates [53]. Intriguingly, multiple NET subtypes can coexist even within the same tumor type; for example, in pancreatic cancer, three positive subtypes defined by PDE4B, IL1B, and TLR2 expression have been identified [54]. In GC, consensus clustering algorithms have stratified patients into two NET‐related subgroups, each exhibiting distinct clinical molecular characteristics, immune microenvironment profiles, and drug responses [51]. Mechanisms of NET formation also vary based on TMEs. In colorectal cancer, analysis of single‐cell RNA sequencing data revealed that the PPIF‐positive neutrophil subpopulation drives mitochondrial ROS‐dependent NET metabolism [55]. In pancreatic cancer, regulation of ROS levels—a balance between NETosis and autophagy—is controlled by the mTOR pathway.

The heterogeneity of NETs carries significant clinical implications. First, it offers considerable prognostic predictive value: in pancreatic cancer, a risk score constructed from four NET‐related genes can predict patient survival and immunotherapy response [56]; similarly, distinct overall survival and disease‐free survival periods are observed in the two GC NET subgroups [57]. More importantly, NET heterogeneity provides opportunities for precision therapeutic targeting. Rather than broadly suppressing NETosis—which may compromise host defense and induce unintended pathologies—targeted interventions can be developed based on genes specifically upregulated in distinct cancer types or NET subtypes. This approach aligns with the multimodal framework by enabling selective disruption of the dominant modality associated with a given tumor, thus achieving more effective and safer NET‐directed therapies. Understanding how compositional heterogeneity shapes the engagement of biomechanical, immunometabolic, and signal‐integration modalities will be essential for translating these insights into clinical practice.

## 3 Mechanisms and Forms of NETosis

### 3.1 Lytic and Non‐lytic Pathways of NET Formation

NETosis occurs through two principal pathways: lytic (classical) NETosis and nonlytic (vital) NETosis. Lytic NETosis is the canonical, ROS‐dependent pathway typically induced by microbial stimuli, cytokines, or phorbol esters [58, 59, 60]. This process involves dismantling the nuclear envelope, chromatin decondensation, and eventual plasma membrane rupture, leading to neutrophil lysis and death [8, 60, 61]. In contrast, nonlytic NETosis is a fast‐acting, NADPH oxidase‐independent process that preserves cell viability. Neutrophils release NETs via vesicular transport without rupturing the plasma membrane, thereby retaining their phagocytic and chemotactic functions [62]. Following NET release, clearance mechanisms such as macrophage‐mediated phagocytosis, DNase I degradation, and autophagy contribute to the removal of NETs [17]. Impaired clearance can lead to the accumulation of NET remnants, fostering chronic inflammation and the formation of protumorigenic niches (Figure 2).

**FIGURE 2:**
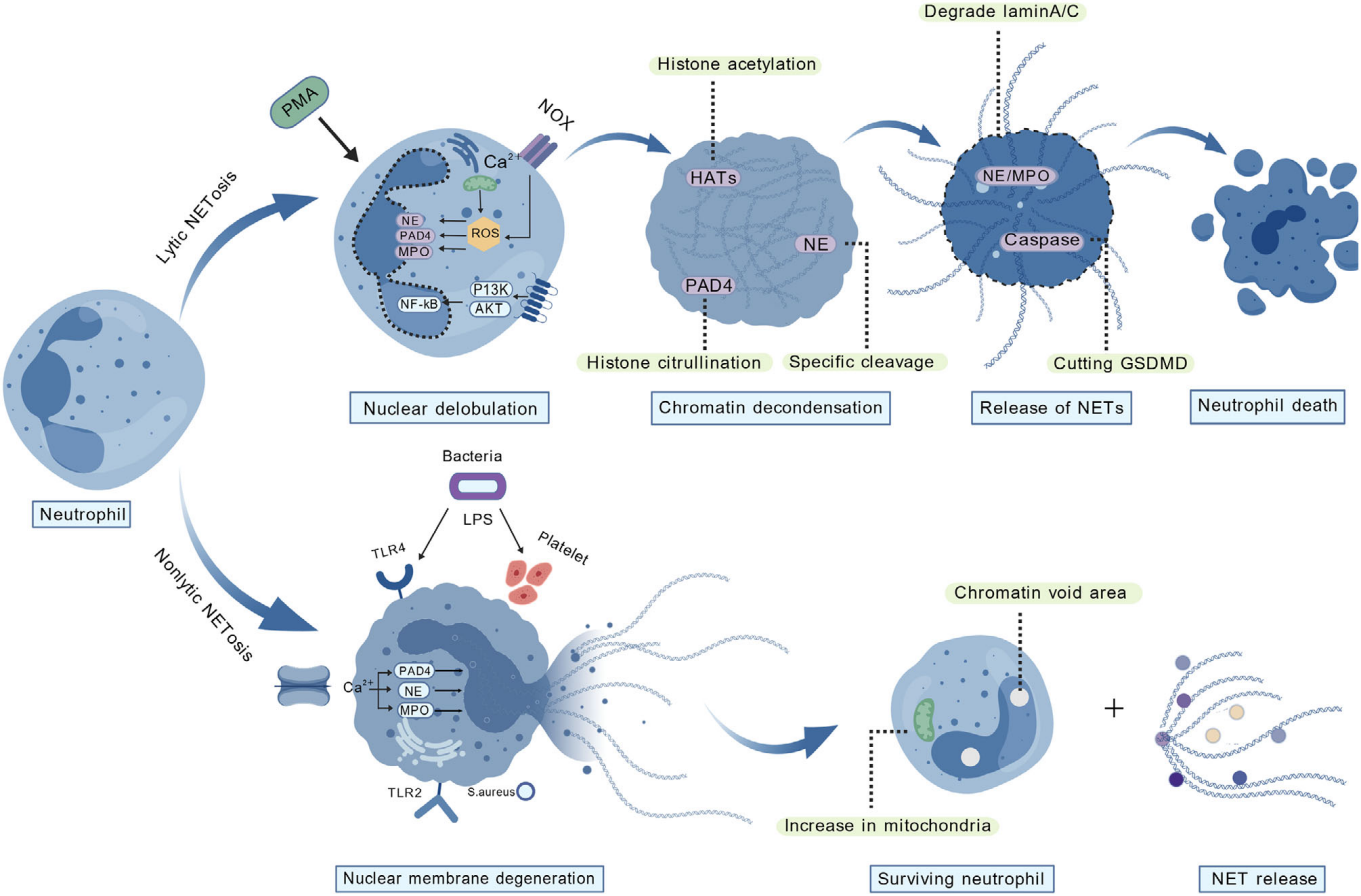
Distinct pathways of NET formation. NETs arise through two primary modes: lytic and nonlytic NETosis. Lytic NETosis is ROS‐dependent and involves nuclear envelope disintegration, chromatin decondensation, and eventual plasma membrane rupture, culminating in neutrophil lysis. Nonlytic NETosis preserves cellular viability; PAD4 activation facilitates nuclear translocation of NE and MPO, enabling chromatin decondensation and vesicular extrusion of NET material without plasma membrane disruption. These enucleated cells may retain limited phagocytic capacity, although their functional longevity remains debated. Both pathways depend on key mediators including MPO, PAD4, and NE. Abbreviations: NE, neutrophil elastase; PAD4, peptidylarginine deiminase 4; ROS, reactive oxygen species; NET, neutrophil extracellular trap; MPO, myeloperoxidase.

The molecular machinery orchestrating NETosis centers on several key mediators. Upon microbial stimulation, phagosomes activate the NOX2 complex, transferring electrons from NADPH to oxygen to produce ROS [63, 64]. These ROS serve as signaling molecules and effectors that damage vesicular and nuclear membranes, while also activating downstream enzymes, including MPO and NE [65, 66]. NE, stored in azurophilic granules, is released into the cytoplasm upon ROS activation, where it binds to F‐actin, induces actin depolymerization, and translocates to the nucleus. Once inside, NE cleaves core histones—particularly H1 and H4—to promote chromatin decondensation [67]. Concurrently, MPO catalyzes halide oxidation, producing hypochlorous acid and other potent oxidants that further facilitate microbial killing and chromatin relaxation [68, 69]. This pathway is clinically validated by observations that patients with chronic granulomatous disease (CGD), who lack functional NADPH oxidase, exhibit defective NETosis [70].

Peptidylarginine deiminase 4 (PAD4) serves as another key regulator. In response to ROS stimulation, PAD4 citrullinates histones H3 and H4, neutralizing the positive charges that mediate electrostatic interactions between histones and DNA. This charge neutralization is essential for large‐scale chromatin decondensation [71, 72]. Pharmacological inhibition of PAD4 reduces NETosis, and PAD4 knockout mice demonstrate impaired NET formation [73]. However, residual NETs can form in PAD4‐deficient contexts, suggesting alternative, PAD4‐independent mechanisms may exist, though their physiological relevance remains unclear [74, 75]. This controversy warrants resolution, especially as PAD4 inhibitors are currently being considered for clinical use. In the context of breast cancer lung metastasis, PAD4 acts as a molecular switch linking tumor‐derived signals, such as IL‐8, to NETosis. By catalyzing histone citrullination and altering chromatin structure, PAD4 promotes NET formation, establishing a prometastatic niche that enhances circulating tumor cell (CTC) adhesion, extravasation, and outgrowth in the pulmonary parenchyma.

Beyond enzymatic mediators, NETs are decorated with coagulation‐related molecules such as von Willebrand factor (vWF), fibronectin, nucleic acids, and histones, which can electrostatically activate platelets [76]. Activated platelets, in turn, amplify further NETosis, creating a self‐reinforcing loop that couples thrombosis with sterile inflammation [77]. Importantly, the molecular machinery of NETosis—encompassing ROS generation, PAD4 activation, and NE/MPO nuclear translocation—functions not merely as an executor of cell death but also as a gatekeeper for signal integration. The intensity and duration of these signals determine whether NETs adopt a predominantly biomechanical, metabolic, or inflammatory phenotype. This determination establishes a direct mechanistic link between NET formation and the functional modalities elaborated in subsequent sections. This unified perspective consolidates the mechanistic foundation of NETosis and provides a coherent entry point for understanding how NETs function as multimodal signal processors within the TME. In the following section, this mechanistic foundation will be built upon to demonstrate how the three functional phenotypes—biomechanical, immunometabolic, and signal‐integrating—operate as interconnected modalities that collectively drive tumor progression.

### 3.2 Clearance of NETs

The mechanisms underlying the clearance of NETs remain unclear. While infection can degrade the DNA component of NETs through DNase I, residual granular proteins and histones may persist, suggesting that other clearance mechanisms—such as macrophage‐mediated phagocytosis, lysosomal degradation, and selective autophagy (e.g., LC3‐associated phagocytosis)—may also contribute to their removal [78]. Additional mechanisms leading to NET clearance include replenishment systems [79], autophagy [80], and macrophage‐mediated clearance [81]. The failure to clear NET remnants may contribute to chronic inflammation and autoimmunity, with new studies suggesting that impaired NET clearance also plays a role in the formation of protumorigenic, immunosuppressive niches.

## 4 The Physiological and Pathological Roles of NETs

Before exploring the tumor‐promoting roles of NETs, it is essential to understand their physiological functions and contributions across a spectrum of nonmalignant diseases. This broader perspective contextualizes the dual nature of NETs—protective in certain settings and pathogenic in others—and reveals that the mechanistic principles governing NET biology are conserved across various disease states. The same molecular machinery that entraps pathogens concurrently drives sterile inflammation in autoimmunity, vascular dysfunction in thrombosis, and tissue remodeling in chronic inflammatory diseases [82]. Understanding these shared mechanisms lays the foundation for the multimodal framework that will be developed in the context of cancer while also highlighting the therapeutic opportunities and challenges that extend beyond oncology.

In addition to their antimicrobial functions, NETs have been implicated in various nonmalignant pathologies. In infectious diseases, uncontrolled NET formation can exacerbate tissue damage and sepsis. In autoimmune disorders, such as SLE and rheumatoid arthritis (RA), NETs act as sources of autoantigens that drive chronic inflammation and contribute to tissue destruction [83]. In thrombosis, NETs provide scaffolds for platelet aggregation and fibrin deposition, linking sterile inflammation to prothrombotic states [84]. These noncancer contexts illustrate the pathological versatility of NETs and offer important insights into their roles in malignancy—especially concerning chronic inflammation, vascular dysfunction, and immune dysregulation. Building on this broader perspective, attention shifts to the TME, where NETs exhibit a context‐dependent duality as both promoters of tumor progression and potential mediators of antitumor responses.

### 4.1 Physiological Functions

NETs represent an evolutionarily preserved component of the innate immune system, primarily functioning to entrap and neutralize pathogenic organisms, including bacteria, fungi, parasites, and viruses [85, 86]. Upon activation by microbial products, neutrophils release chromatin fibers decorated with histones and granular enzymes such as NE, MPO, and bactericidal permeability‐increasing protein [87, 88, 89]. These web‐like structures immobilize microorganisms through electrostatic interactions, facilitating high local concentrations of antimicrobial peptides and proteases that exert cytotoxic effects and degrade microbial virulence factors [90, 91]. Empirical research confirms that NETs effectively trap and kill various pathogens, including Staphylococcus aureus, Escherichia coli, and Candida albicans [6, 92]. However, uncontrolled or persistent NET release can inflict significant collateral damage on host tissues. ECs and various organs, such as the liver, can suffer from injury associated with NET‐derived proteases and histones [93, 94, 95]. Consequently, the mechanisms safeguarding against infection may, under pathological conditions, contribute to sterile inflammation, microvascular thrombosis, and organ dysfunction.

Beyond their antimicrobial functions, NETs also contribute to immune homeostasis. Neutrophils rapidly infiltrate inflamed tissues, and following pathogen clearance, undergo apoptosis to stabilize the environment [96]. Cytokines and chemokines are proinflammatory and can be sequestered and enzymatically degraded by NETs into smaller, less inflammatory molecules, thereby inhibiting the escalation of inflammatory responses [14, 60, 97, 98, 99]. Furthermore, NETs can stimulate Type I interferon production through the activation of plasmacytoid dendritic cells (DCs) via TLR9, thereby mediating connections between innate and adaptive immunity [100, 101]. They also engage complement components such as C3 and factor B, which amplify local inflammation and create prothrombotic surfaces through C5a and membrane attack complex MAC deposition [102, 103]. Thus, NETs function as a double‐edged sword—providing essential frontline antimicrobial protection while having the potential to provoke tissue injury, sustained inflammation, and immune intolerance. These dual roles form the basis for their contributions to cancer‐related inflammation, metastatic niche formation, and therapeutic resistance.

Beyond their protective role in host defense, NETs are implicated in diverse pathological states. These pathologies are not random; rather, they can be systematically categorized according to the multimodal framework. At the core, NETosis functions as a multimodal signal processor, generating three distinct outputs: biomechanical, immunometabolic, and signal‐integration modalities.

### 4.2 Pathological Functions

Beyond their protective role in host defense, NETs contribute to a wide spectrum of nonmalignant pathologies through distinct mechanistic modalities (Figure 3). The biomechanical modality is driven by the physical DNA‐protein scaffold of NETs and the proteolytic activity of NE and MMP‐9. These biomechanical outputs underlie thrombogenesis by trapping platelets and red blood cells, promoting clot formation and vascular occlusion. Concurrently, NE and MMP‐9 degrade ECM components, including collagen and laminin, resulting in tissue destruction and vascular damage that facilitate further pathological remodeling [104]. These biomechanical properties create physical tracks that contribute to tumor cell migration and metastasis.

**FIGURE 3:**
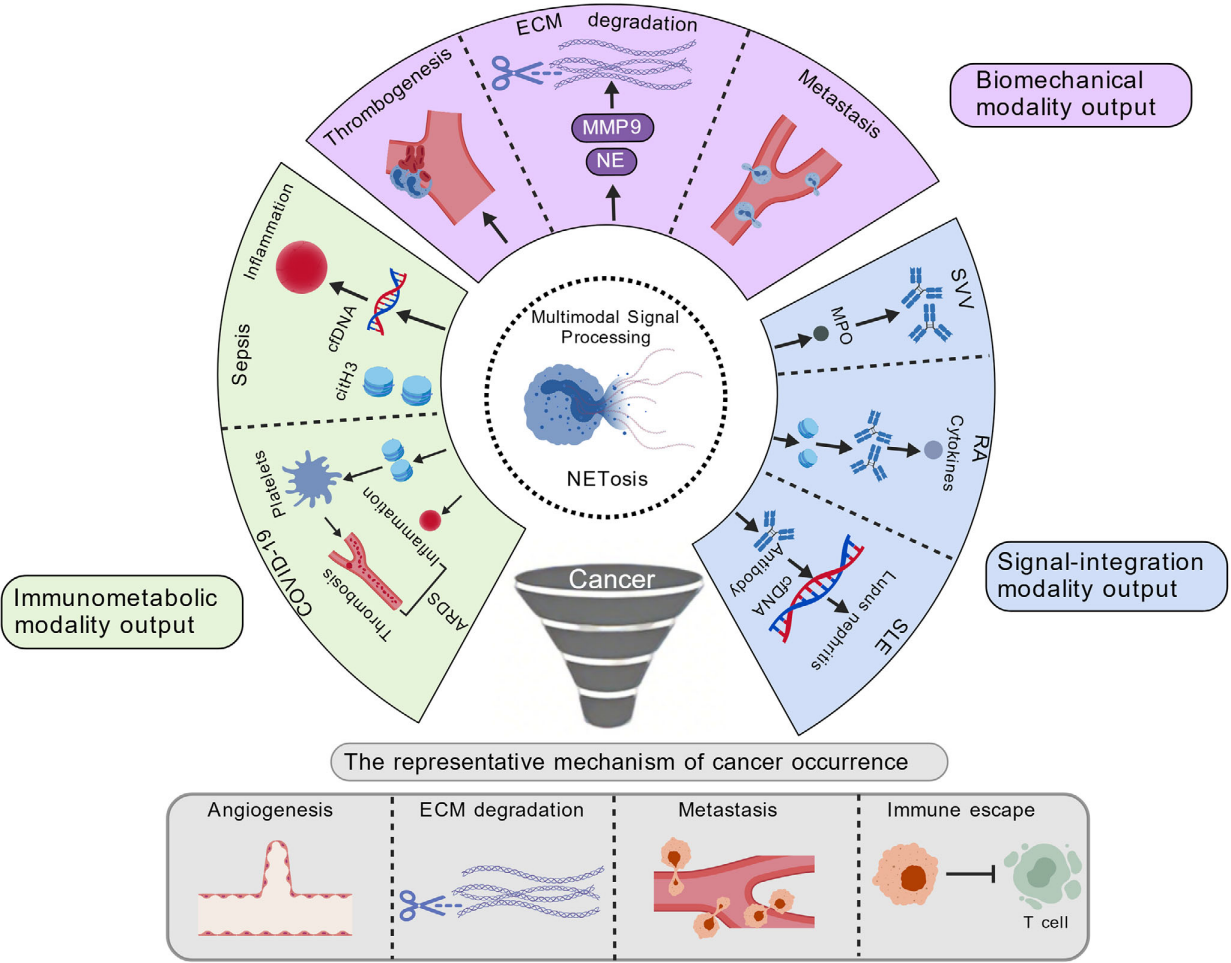
The pathological continuum of NETs as a multimodal signal‐processing network. The central core represents NETosis as a multimodal processor, with surrounding pathologies categorized into three mechanistic sectors. The biomechanical sector includes thrombosis and ECM degradation, the immunometabolic sector includes sepsis, ARDS, and COVID‐19‐related inflammation, and the signal‐integration sector includes SLE, RA, and SVV. The bottom funnel illustrates that chronic inflammation and hypoxia push these modalities beyond a functional threshold, leading to their synergistic convergence. Cancer progression is shown here as a representative endpoint of this convergence, with the bottom panel depicting four key mechanisms including angiogenesis, ECM degradation, metastasis, and immune escape. Importantly, this framework extends beyond oncology and provides a universal mechanistic lens for understanding NETs‐driven pathology across inflammatory, thrombotic, and autoimmune diseases. Abbreviations: NE, neutrophil elastase; MPO, myeloperoxidase; DAMPs, damage‐associated molecular patterns; ANCA, antineutrophil cytoplasmic antibody; ACPA, anticitrullinated protein antibody; citH3, citrullinated histone H3; cfDNA, cell‐free DNA; SLE, systemic lupus erythematosus; NETs, neutrophil extracellular traps; ARDS, acute respiratory distress syndrome.

The immunometabolic modality emerges from the substantial production of ROS during NETosis, along with hypoxia‐driven glycolytic reprogramming. This metabolic dysregulation generates a hostile microenvironment characterized by oxidative stress and lactate accumulation. This modality is central to the pathogenesis of sepsis and acute respiratory distress syndrome, where uncontrolled NET formation exacerbates systemic inflammation and multiorgan dysfunction. In severe COVID‐19, activated platelets and NETs form a self‐amplifying loop of inflammation and thrombosis, further driving pulmonary injury and hypercoagulable states [105]. The acidic and hypoxic conditions created by this metabolic reprogramming compromise immune cell function, fostering a permissive environment for chronic inflammatory diseases.

The signal‐integration modality involves the spatial concentration of autoantigens such as cfDNA, citrullinated histones, and MPO on the NET scaffold. This concentrated presentation serves as a platform for activating autoreactive B and T cells. In SLE, NET‐derived cfDNA triggers anti‐dsDNA antibody production, leading to immune complex deposition and lupus nephritis [106]. In RA, citrullinated histones and other NET‐associated antigens stimulate the production of anticitrullinated protein antibodies, sustaining chronic joint inflammation and bone destruction [107]. In systemic vasculitis, MPO exposure on NETs induces antineutrophil cytoplasmic antibody production, further amplifying neutrophil activation and vascular injury. These self‐perpetuating inflammatory cycles exemplify how the signal‐integration modality drives chronic autoimmune pathology.

### 4.3 Transition To Malignancy

While the nonmalignant pathologies described above arise from distinct modalities, they do not operate in isolation. Continuous chronic inflammation, persistent hypoxia, and progressive tissue stiffening within the pathological microenvironment collectively push the multimodal network beyond a functional threshold. Once this threshold is exceeded, the three modalities converge synergistically to drive malignant progression (Figure 3).

The lower panel of Figure 3 further illustrates the representative mechanisms of cancer development resulting from this multimodal convergence. Angiogenesis is fueled by HIF‐1α‐driven vascular endothelial growth factor (VEGF) release from the immunometabolic hub, while ECM degradation is executed by the proteolytic activity of NE and MMP‐9 within the biomechanical scaffold [108]. Metastasis is guided by the physical migration tracks provided by the NET scaffold, and immune escape is mediated through immunosuppressive signals concentrated on the signal‐integration platform. These processes do not occur sequentially; rather, they intertwine to establish a protumorigenic microenvironment that supports tumor growth, invasion, and dissemination.

Thus, cancer represents not merely an isolated pathological state but the culmination of a multimodal pathological continuum orchestrated by NETs. This systems‐level integration of biomechanical, immunometabolic, and signal‐integration pathways sets the stage for the detailed tumor‐promoting mechanisms elaborated in subsequent sections.

## 5 Tumor‐Promoting Roles of NETs

Researchers have often characterized the tumor‐promoting functions of NETs based on the stages of cancer they impact—initiation, proliferation, invasion, and metastasis. This stage‐based perspective has generated an extensive list of NET‐associated molecules and effects, yet it does not elucidate the organizational principles that govern the diverse outcomes produced by NETs [109]. Moving beyond this descriptive approach, a framework is proposed wherein NETs drive tumor growth through three interconnected functional modalities: biomechanical scaffolds that physically remodel the tumor environment and activate mechanotransduction; immunometabolic hubs that reprogram cellular energy utilization and link these changes to immune suppression; and signal‐integration platforms that concentrate diverse microenvironmental cues for synergistic effects. These modalities do not operate independently; instead, they engage in dynamic crosstalk through core interaction nodes that integrate signals across modalities.

Core interaction nodes serve as linchpins in this network. HIF‐1α resides at the intersection of the biomechanical scaffold and the immunometabolic hub: mechanical signals such as tissue stiffness activate FAK, stabilizing HIF‐1α, while hypoxic conditions directly induce HIF‐1α to drive glycolytic reprogramming [110, 111]. Conversely, the integrin–FAK axis links the biomechanical scaffold and the signal‐integration platform: integrin engagement by NET‐bound adhesion ligands triggers FAK phosphorylation, initiating cytoskeletal remodeling and amplifying signaling from colocalized growth factor receptors, such as epidermal growth factor receptor (EGFR) and c‐Met [112]. These nodes ensure that signals from different modalities converge, amplify, and produce coherent functional outputs.

NETs sense contextual signals through specific surface receptors on tumor cells and stromal cells. Mechanical stress, such as ECM stiffness, is detected via integrins; metabolic stress, including hypoxia and lactate, is sensed through HIF‐1α‐dependent pathways; and inflammatory cues like IFN‐γ, transforming growth factor‐beta (TGF‐β), and IL‐8 are recognized by TLRs and cytokine receptors. Once engaged, these receptors initiate coordinated activation across the three modalities [52, 113, 114]. For example, hypoxia simultaneously activates HIF‐1α‐driven glycolysis within the immunometabolic hub and enhances FAK‐mediated mechanotransduction within the biomechanical scaffold, while TGF‐β binding to its receptor upregulates integrin expression within the signal‐integration platform and promotes M2 macrophage polarization within the immunometabolic hub [115, 116]. This context‐dependent multimodal integration enables NETs to execute either protumorigenic or antitumorigenic outputs based on the intensity and combination of inputs.

Transitioning from descriptive cataloging to a systems‐level understanding necessitates recognizing that NETs constitute an integrated signal‐processing network rather than mere collections of effector molecules. The three modalities—biomechanical, immunometabolic, and signal‐integration—are linked by core interaction nodes, such as HIF‐1α and the integrin–FAK axis, which convert diverse microenvironmental cues into amplified, context‐specific functional outputs. This integrated perspective elucidates how NETs can promote tumor progression under chronic inflammation and hypoxia yet exert antitumor effects under acute IFN‐γ‐rich conditions.

### 5.1 NETs As Biomechanical Scaffolds

Before being recognized as sources of proteases or cytokines, NETs exist as physical objects—macromolecular networks with distinct biomechanical properties [117]—where their DNA‐protein fibers create a rigid, elastic scaffold that interfaces directly with tumor cells and stromal components. This physical interface is active, sensed by cells, and transduced into biochemical signals regulating proliferation, migration, and survival.

#### 5.1.1 Integrin–FAK Mechanotransduction

The DNA‐histone backbone of NETs engages integrin receptors on tumor cells, triggering FAK autophosphorylation and downstream signaling cascades such as Rho/ROCK, mitogen‐activated protein kinase (MAPK), and PI3K–Akt, which drive cytoskeletal remodeling, gene transcription, and cell survival [118, 119] (Figure 4). NETs function as dynamic hubs that integrate immune and mechanical cues to drive tumor invasion through the integrin–FAK signaling axis [120]. The three‐dimensional network formed by NE, MPO, and the histone‐coated DNA backbone exhibits both stiffness and elasticity—a defining property of the biomechanical scaffold modality. This structure presents a noncanonical ECM that engages integrin receptors [121]. Unlike classical ECM components such as collagen, laminin, and fibronectin—which are structured, static, and primarily homeostatic—NET‐derived scaffolds are dynamic, inflammation‐induced structures that simultaneously deliver mechanical cues alongside a rich payload of proteases, chemokines, and damage‐associated molecules. This unique combination enables NETs to activate integrin signaling in a manner that is both mechanically and biochemically reinforced, resulting in downstream effects that are more robust and sustained than those induced by classical ECM alone.

**FIGURE 4:**
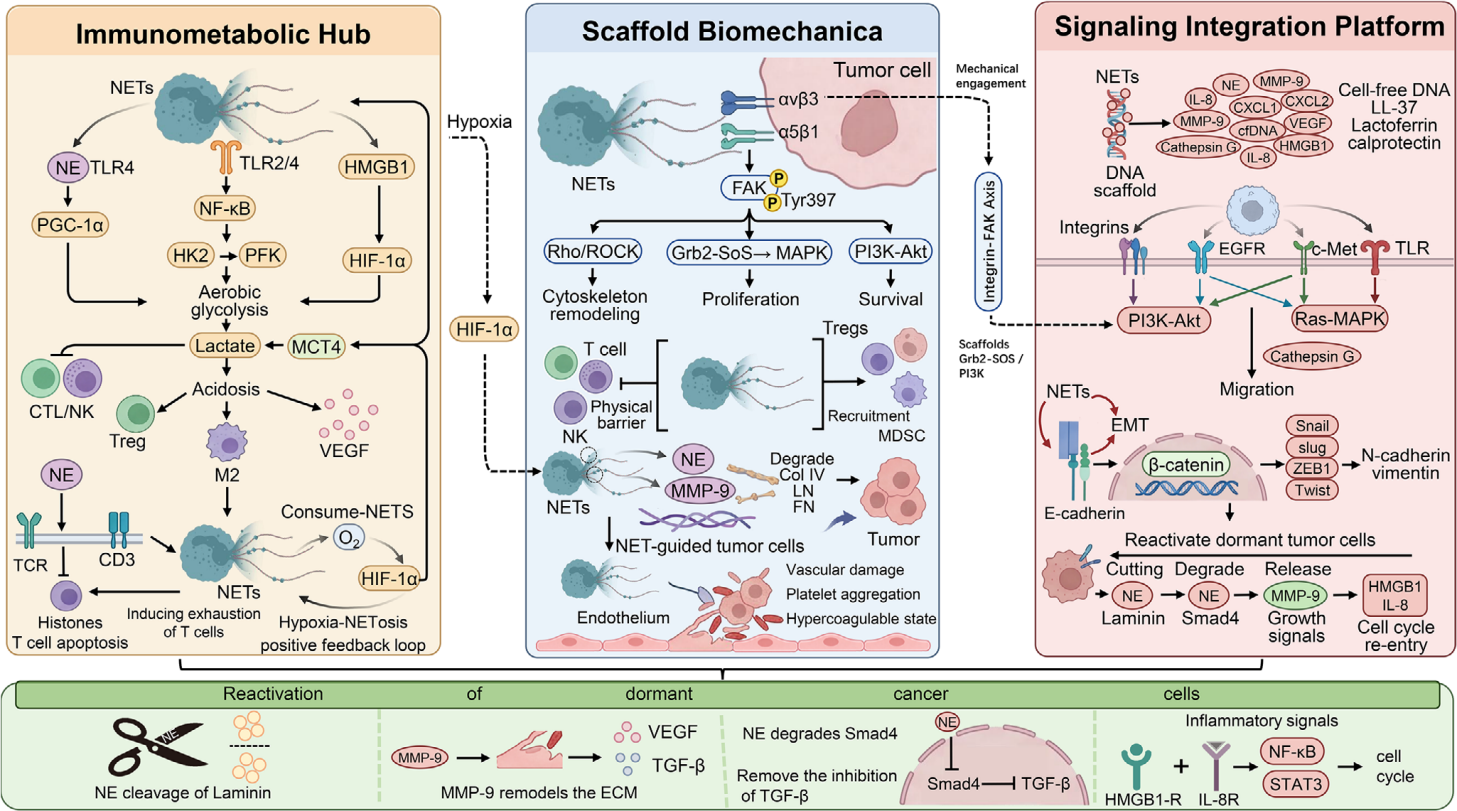
NETs as a signal‐integration platform driving multimodal tumor progression. Diverse NETs‐derived ligands including IL‐8, HMGB1, HGF, EGF, NE, and MPO converge on tumor cell surface receptors such as CXCR1/2, c‐Met, EGFR, and TLR4 to form a localized signal‐integration platform. These spatially concentrated cues activate shared downstream pathways including the RAS–RAF–MEK–ERK axis, PI3K–AKT–mTOR signaling, NF‐κB, and JAK–STAT cascades, resulting in synergistic oncogenic activation. The ultimate functional outputs are categorized within the multimodal framework, with growth and proliferation representing biomechanical modal outputs, while antiapoptosis and immune escape correspond to immunometabolic modal outputs. This schematic highlights the central role of the signal‐integration platform in converting heterogeneous microenvironmental signals into coordinated tumor‐promoting responses. Abbreviations: HIF‐1α, hypoxia‐inducible factor 1‐alpha; FAK, focal adhesion kinase; HMGB1, high mobility group box 1; NETs, neutrophil extracellular traps; TLR, Toll‐like receptor; HMGB1, high‐mobility group box 1; EMT, epithelial–mesenchymal transition; Col IV, Collagen Type IV; LN, laminin; FN, fibronectin.

Among integrin receptors, αvβ3 and α5β1 on tumor cell membranes specifically bind to adhesion ligands embedded within NET structures [122]. This interaction facilitates integrin clustering and the formation of nascent adhesion sites, which subsequently recruit adaptor proteins such as talin, tensin, and vinculin to generate mature focal adhesion complexes [123]. These complexes serve as key signaling platforms where FAK undergoes autophosphorylation at Tyr397, marking its activation [124, 125]. Activated FAK transmits downstream signaling through Rho family GTPases, activating ROCK, which phosphorylates and suppresses myosin light chain phosphatase, resulting in sustained myosin light chain phosphorylation [126]. This enhancement of actomyosin contractility promotes cytoskeletal remodeling and directional cell migration. Simultaneously, FAK acts as a scaffold for the Grb2–SOS complex, activating Ras and triggering the MAPK pathway, which promotes the transcription of genes associated with tumor cell growth, survival, and invasion [127]. Additionally, phosphorylated FAK specifically interacts with the p85 subunit of PI3K, stimulating Akt signaling and enhancing cellular plasticity within the invasive microenvironment [128].

Crucially, mechanical and immune signaling processes are not distinct but are mutually regulated through a feedback loop. NETs increase IL‐8 sensitivity in tumor cells via the integrin–FAK pathway: FAK drives elevated expression of CXCR1 and CXCR2 [129], while IL‐8 signaling reciprocally activates integrins, increasing their ligand affinity and establishing a self‐reinforcing circuit that sustains chemotactic and adhesive behaviors [130]. FAK also interacts extensively with the TGF‐β/Smad pathway, directly phosphorylating Smad proteins to enhance their transcriptional activity [131]. While TGF‐β promotes the transition of epithelial cells toward an invasive mesenchymal phenotype through epithelial–mesenchymal transition (EMT), the NET–FAK axis provides persistent mechanical and survival cues that stabilize this mesenchymal state and prevent mesenchymal–epithelial transition [132]. Concurrently, the original ECM degrades due to NET‐related proteases while newly deposited ECM components are produced by tumor cells and cancer‐associated fibroblasts activated through FAK‐dependent pathways [133, 134]. This process not only enhances the structural integrity of the invasive front but also remodels the peritumoral stroma into a migration‐permissive matrix, effectively creating prealigned pathways for collective or single‐cell invasion.

Overall, these findings position NETs as mechanochemical constructors that integrate inflammatory and biomechanical cues, finely tuning the invasive apparatus of malignant cells. Through the integrin–FAK axis, NETs convert mechanical cues into a cascade of proinvasive signals that promote tumor cell migration, proliferation, and survival, establishing the biomechanical scaffold as a key driver of tumor progression.

#### 5.1.2 Physical Barrier Function and Immune Exclusion

Beyond activating signaling, the NET meshwork serves as a physical barrier that impedes immune cell infiltration [135]. Dense perivascular NET deposits exclude cytotoxic T lymphocytes and natural killer (NK) cells from tumor nests, creating immune‐privileged zones where tumor cells can proliferate unchecked [136]. This physical exclusion synergizes with biochemical immunosuppression; the same NET fibers that obstruct lymphocyte access also present chemokines that recruit regulatory T cells and myeloid‐derived suppressor cells [137].

#### 5.1.3 ECM Remodeling and Migration Tracks

NET‐associated proteases, particularly NE and MMP‐9, actively degrade ECM components, including Type IV collagen, laminin, and fibronectin. This degradation serves a dual purpose by clearing physical obstacles to tumor cell migration and liberating matrix‐bound growth factors that further stimulate invasion [138]. The resulting ECM fragments also act as chemotactic signals, guiding tumor cells along NET‐defined paths [135]. Time‐lapse imaging has revealed tumor cells migrating along NET fibers, utilizing them as preformed “highways” for collective or single‐cell invasion. Clinically, neutrophils from GC patients exhibit enhanced NETosis correlated with increased invasiveness and angiogenesis, whereas DNase I‐mediated NET degradation suppresses NE and MMP activity while reducing metastatic potential.

MMPs are known to stimulate integrin–FAK signaling, facilitating cytoskeletal remodeling and metastatic dispersal mechanistically [139, 140]. Invasion necessitates the destruction of basement membrane components. MMP‐2 and MMP‐9 preferentially degrade Collagen IV, a major constituent of the basement membrane [141]. Concurrently, NET‐derived NE and MMP‐9 cleave laminin, further weakening the basement membrane and enhancing trans‐barrier migration [14]. This active proteolysis positions NETs as active agents of early metastatic dissemination.

NET‐mediated ECM remodeling is not merely a proteolytic event; it represents a synergistic output of the multimodal network. The biomechanical scaffold provides physical migration tracks, the signal‐integration platform supplies chemotactic cues through released VEGF and TGF‐β, and the immunometabolic hub supports the process with local glycolytic energy. This coordinated action signifies a core proinvasive output of the NET network.

#### 5.1.4 Vascular Disruption and Thrombosis

The biomechanical influence of NETs extends to the vasculature. NETs deposited on the endothelial surface disrupt vascular integrity through several mechanisms: histones directly damage ECs, NET‐associated proteases degrade junctional proteins, and the NET meshwork creates turbulent flow that promotes platelet activation. The outcome is a hyperpermeable, prothrombotic vasculature that facilitates tumor cell intravasation while simultaneously shielding CTCs from shear stress and immune surveillance.

The neovasculature pathology driven by NETs fails to provide adequate perfusion and results in a delicate and porous intravascular system prone to thrombosis and metastatic cross‐linking. Proteolytic and oxidative events triggering vascular remodeling expose procoagulant surfaces and endothelial damage, promoting thrombus development. NETs create a structural and biochemical framework fostering thrombus formation while compromising vascular integrity, thereby coupling thrombosis with metastatic dissemination. In thrombi, the DNA fibers of NETs create a reticular scaffold that traps platelets and red blood cells, facilitating fibrin deposition and stabilizing clot structure. Platelet TLR2/4 can be directly activated by histones H3 and H4 exposed on NETs, leading to platelet aggregation, degranulation, and activation [142, 143]. Concurrently, NE degrades anticoagulant proteins such as thrombomodulin and tissue factor pathway inhibitor, shifting the hemostatic balance toward a hypercoagulable state.

The abuse of the endothelium represents a critical initial stage of NET‐mediated thrombosis. Cytotoxicity associated with NETs exposes subendothelial collagen and vWF, facilitating platelet adhesion through glycoprotein Ib–vWF and integrin α2β1–collagen interactions [144, 145]. Upon adhesion, platelets release ADP and thromboxane A_2_ (TXA_2_), activating αIIbβ3 integrins, which promote fibrinogen cross‐linking and stabilize platelet aggregates [146, 147, 148]. Concurrently, ROS generated during NETosis exacerbate oxidative stress on ECs, leading to apoptosis and further exposure of procoagulant surfaces. Collectively, these events establish a self‐amplifying thromboinflammatory circuit driven by reciprocal interactions among platelets, NETs, and ECs.

Clinical observations from autoimmune disorders, such as antiphospholipid syndrome (APS), highlight this cycle. Anti‐β_2_ glycoprotein I antibodies promote NETosis via ROS production by NADPH oxidase, while NET‐derived DNA and histones stimulate ECs and monocytes to express tissue factor, prompting thrombin production and fibrinogenesis [149, 150]. This creates a positive feedback loop in which immune activation, endothelial destruction, and NETosis uphold intravascular thrombosis. Beyond hemostasis, NETs serve as key activators of hematogenous tumor dissemination. Their DNA‐protein scaffolds encapsulate CTCs, protecting them from shear stress and NK cell‐mediated clearance, while concurrently enhancing adhesion to activated endothelium [151]. Additionally, NET‐related histones and cytokines compromise endothelial integrity, increase vascular permeability, and provide chemotactic signals that facilitate metastatic seeding [152]. Therefore, NETs act as dual effectors, linking thrombosis to vascular leakage and creating a direct connection between coagulation and the metastatic cascade [153, 154]. The biomechanical functions of NETs do not operate in isolation; mechanical signals transduced through integrin–FAK interactions converge with metabolic and inflammatory pathways, forming a mechano–metabolic–inflammatory axis that amplifies protumorigenic outputs [110, 155]. As discussed in the following section, the NET scaffold not only provides physical cues but also serves as a hub for metabolic reprogramming.

This prothrombotic function extends beyond oncology; similar NET‐driven mechanisms contribute to deep vein thrombosis and APS, suggesting therapeutic strategies targeting NET‐platelet interactions may have broad applicability.

### 5.2 NETs as Immunometabolic Hubs

Beyond their physical presence, NETs significantly reprogram cellular metabolism within the TME. By consuming oxygen, releasing reactive species, and presenting metabolically active enzymes, NETs create a distinct metabolic niche that suppresses immune cells while favoring tumor cell metabolism [136, 156]. This section establishes NETs as central nodes in the tumor's metabolic circuitry.

#### 5.2.1 Induction of Glycolytic Reprogramming

NETs drive metabolic reprogramming through multiple convergent pathways that operate in parallel with and often amplify tumor‐intrinsic metabolic adaptations. NET‐associated NE activates TLR4–peroxisome proliferator‐activated receptor gamma coactivator 1‐alpha (PGC‐1α) signaling in tumor cells, promoting mitochondrial biogenesis and oxidative metabolism. In macrophages, NET components engage TLR2/4–NF‐κB signaling, upregulating glycolytic enzymes such as hexokinase 2 (HK2) and phosphofructokinase (PFK) [157]. This glycolytic switch—the Warburg effect—is further amplified by NET‐released high mobility group box 1 (HMGB1) (Figure 4) [158]. Importantly, while tumor cells intrinsically undergo metabolic reprogramming driven by oncogenic mutations, NETs function as an external microenvironmental driver that reinforces and sustains this metabolic shift through paracrine signaling. This creates a glycolysis‐dominant landscape where glucose is preferentially fermented to lactate, even in the presence of oxygen. This metabolic configuration supports rapid tumor cell proliferation while simultaneously generating an environment hostile to effector immune cells.

Beyond these direct metabolic effects, NETs promote malignant cell proliferation through mechanisms distinct from tumor‐autonomous metabolic changes. In chronic lymphocytic leukemia, NETs induce CD5^+^ B‐cell expansion by stimulating the release of IL‐1β, IL‐6, and tumor necrosis factor‐alpha (TNF‐α) via NF‐κB–dependent pathways [159, 160]. In pancreatic ductal adenocarcinoma, extracellular DNA released from NETs activates pancreatic stellate cells, driving desmoplastic fibrosis and generating a stiff, growth‐permissive microenvironment [161, 162, 163]. NET‐related molecules also function as potent facilitators of tumor cell growth: HMGB1 released by NETs stimulates TLR4 and receptor for advanced glycation end products on cancer cells, promoting survival and proliferation [164]; NE enhances colorectal cancer growth through the TLR4–PGC‐1α axis, driving mitochondrial biogenesis and the release of IL‐1β and IL‐6, as well as metabolic activation [165, 166]; and prostaglandin E2 released by NETs promotes non‐small cell lung cancer cell growth through autocrine inflammatory pathways [167]. In vitro studies confirm that NETs or their components significantly stimulate the growth of pancreatic cancer, colon cancer, and hepatocellular carcinoma cell lines [168]. The underlying mechanism primarily involves synergistic activation of the EGFR/c‐Met–PI3K/AKT–mTOR and RAS–RAF–MEK–ERK pathways, driving cell cycle progression through heightened expression of cyclin D1–CDK4/6 complexes and reduced activity of cyclin‐dependent kinase inhibitors such as p27, all contributing to G1/S phase transition and enhanced proliferative capacity (Figure 4) [169, 170, 171].

Collectively, these findings demonstrate that NETs do not merely trigger genetic and inflammatory alterations that promote tumor growth; they also actively maintain the proliferative impetus of malignant cells by integrating external inflammatory signals with tumor‐intrinsic metabolic programs [172]. NETs thus function as critical bridges linking oxidative stress, inflammation, and metabolic adaptation, positioning them as key integrators of tumor initiation and progression. Targeting the crosstalk between NETs and tumor cells therefore represents a promising therapeutic strategy for adjuvant cancer treatment.

#### 5.2.2 Lactate Compartmentalization and Acidic Microenvironment

The high glycolytic flux induced by NETs generates significant amounts of lactate, which is exported by cells via monocarboxylate transporter 4 (MCT4). As lactate accumulates, it acidifies the TME, leading to profound immunosuppressive consequences. Acidic conditions directly impair the function of cytotoxic T cells and NK cells, promote M2 polarization of tumor‐associated macrophages, enhance regulatory T cell stability and suppressive activity, and induce VEGF expression, thereby linking metabolism to angiogenesis [173]. Importantly, lactate itself promotes further NETosis, creating a self‐amplifying metabolic loop [174]. In addition to immune modulation, NETs connect inflammation to metabolic reprogramming: they stimulate macrophages to release IL‐1β and TNF‐α, which activate NF‐κB and upregulate glycolytic enzymes such as HK2 and PFK, with TNF‐α also enhancing glycolysis through AP‐1 [175, 176]. The accelerated conversion of pyruvate to lactate results from increased lactate dehydrogenase A activity, while lactate is extruded via MCT4, further acidifying the microenvironment and intensifying immune suppression [177].

This metabolic imbalance is exacerbated by the physical structure of NETs. Dense NET networks consume large amounts of oxygen, creating localized hypoxia along with infiltrating immune cells [178]. Hypoxia stabilizes HIF‐1α in neutrophils, which subsequently upregulates glycolytic genes to sustain ATP production during NETosis and enhances transcription of NE and MPO, promoting further NET formation. This establishes a vicious positive feedback cycle: NET formation drives hypoxia and inflammation, which in turn stimulate further NET release [179, 180]. HIF‐1α also activates NE and MPO simultaneously, linking metabolic stress to sustained NETosis (Figure 4).

Lactate compartmentalization and the resulting acidic microenvironment exemplify the interplay of all three modalities: the immunometabolic hub produces lactate via glycolysis, the biomechanical scaffold exacerbates glycolysis through physical hypoxia, and the signal‐integration platform modifies NET components via histone lactylation, altering their immunoregulatory signals. This cooperative network tightly couples metabolic reprogramming with immune suppression, serving as a hallmark output of the protumorigenic NET ecosystem. Beyond metabolic reprogramming, NETs exert their protumorigenic influence through a third critical function: the spatial concentration and synergistic integration of diverse signaling molecules. This signal‐integration capacity transforms NETs from passive scaffolds into active signaling hubs that amplify oncogenic pathway activation.

#### 5.2.3 Amino Acid Depletion and Metabolic Starvation of Immune Cells

NETs present Arg‐1 on their DNA scaffold, depleting microenvironmental l‐arginine, which is essential for T cell proliferation and effector function. Arginine starvation induces G1 cell cycle arrest in T cells and downregulates CD3ζ chain expression, impairing T cell receptor (TCR) signaling. Similarly, NET‐associated T cells, which are critical to adaptive immunity, also become obstructed by NETs. NE from NETs cleaves components of the TCR/CD3 complex, including the CD8 coreceptor, hindering T cells from recognizing antigens and transmitting signals [181]. Consequently, even in the presence of substantial tumor antigens, T cells cannot obtain the signals necessary to activate. Furthermore, high levels of NETs or their principal components, especially histones, can directly kill T cells [49]. These components induce a form of cell death called apoptosis through mitochondrial pathways or death receptors [182], leading to the loss of functional T cells within the tumor area.

#### 5.2.4 Hypoxia–NETosis Positive Feedback

NETs consume oxygen during ROS production via NADPH oxidase, resulting in local hypoxia that stabilizes HIF‐1α in neutrophils [178]. Within this multimodal framework, HIF‐1α acts as a central node of the immunometabolic hub by upregulating glycolytic genes, such as GLUT1, HK2, and PFKFB3, to sustain ATP production during NETosis, thereby linking metabolic stress to sustained NET release [180]. Simultaneously, HIF‐1α directly enhances the transcription of NE and MPO, two dual‐modal effectors involved in both the immunometabolic hub and the biomechanical scaffold. Specifically, NE and MPO contribute to the immunometabolic hub through Arg‐1‐mediated arginine depletion and lactate metabolism, while also supporting the biomechanical scaffold by degrading ECM components and establishing migration tracks. This arrangement creates a self‐amplifying cycle: hypoxia drives HIF‐1α, which boosts both glycolytic energy supply and the production of NE and MPO, thereby reinforcing the immunometabolic hub while priming the biomechanical scaffold for matrix remodeling (Figure 4).

Importantly, PAD4 serves as the master regulator of NETosis, yet its role within the multimodal framework extends beyond merely driving chromatin decondensation. PAD4‐mediated histone citrullination stabilizes the biomechanical scaffold by altering chromatin charge density and fiber rigidity, and it also participates in the immunometabolic hub through emerging links to histone lactylation—a modification that affects NET‐associated proteins and their immunostimulatory properties. Consequently, the hypoxia–NETosis positive feedback loop represents not a linear mechanistic cascade but rather a systems‐level integration of the immunometabolic and biomechanical modalities, where ROS, PAD4, NE, and MPO collectively orchestrate a protumorigenic network.

This hypoxia–NETosis feedback loop is not exclusive to tumors; it has been identified in pulmonary fibrosis, ischemic stroke, and chronic obstructive pulmonary disease, reinforcing the notion that hypoxia‐driven NET formation constitutes a conserved pathological mechanism across various disease contexts.

### 5.3 NETs as Signal‐Integration Platforms

A distinctive function of NETs lies in their capacity to concentrate and spatially organize diverse signaling molecules. By anchoring proteases, chemokines, growth factors, and damage‐associated molecules to a stable DNA scaffold, NETs create signaling hotspots where tumor cells receive simultaneous and synergistic inputs. This spatial organization transforms NETs from passive collections of molecules into active signaling hubs.

#### 5.3.1 Spatial Concentration of Signaling Molecules

The DNA backbone of NETs functions as a scaffold that binds and retains a diverse array of effectors, including proteases, chemokines, growth factors, damage‐associated molecules, and antimicrobial peptides [183]. This concentration results in localized microenvironments where signaling molecule concentrations far exceed those found in the bulk interstitial fluid. For a tumor cell adhering to a NET fiber, the local concentration of NE or TGF‐β can be orders of magnitude higher than in other tumor regions. Proteins stored in neutrophil granules are released during NETosis and associate with the DNA backbone; for example, calprotectin sequesters zinc to inhibit microbial growth, while lactoferrin chelates iron to disrupt bacterial biofilms—functions that may also contribute to microenvironmental remodeling within the tumor context [184].

#### 5.3.2 Synergistic Receptor Activation

Spatial concentration enables receptor synergy, allowing a tumor cell adhering to a NET to simultaneously engage several receptor systems. Specifically, it interacts with integrins through NET adhesion ligands, EGFR via NET‐associated ligands or NE‐mediated transactivation, c‐Met through HMGB1 or HGF presented on NETs, and TLRs through NET DNA, HMGB1, and histones. These receptors do not signal independently; they converge on shared downstream pathways—particularly the PI3K–Akt and Ras–MAPK pathways—producing synergistic effects on proliferation, survival, and migration. For instance, coactivation of EGFR and c‐Met on NETs leads to stronger and more sustained ERK phosphorylation compared with activation of either receptor alone, thereby driving cyclin D1 expression and promoting G1/S progression (Figure 5).

**FIGURE 5:**
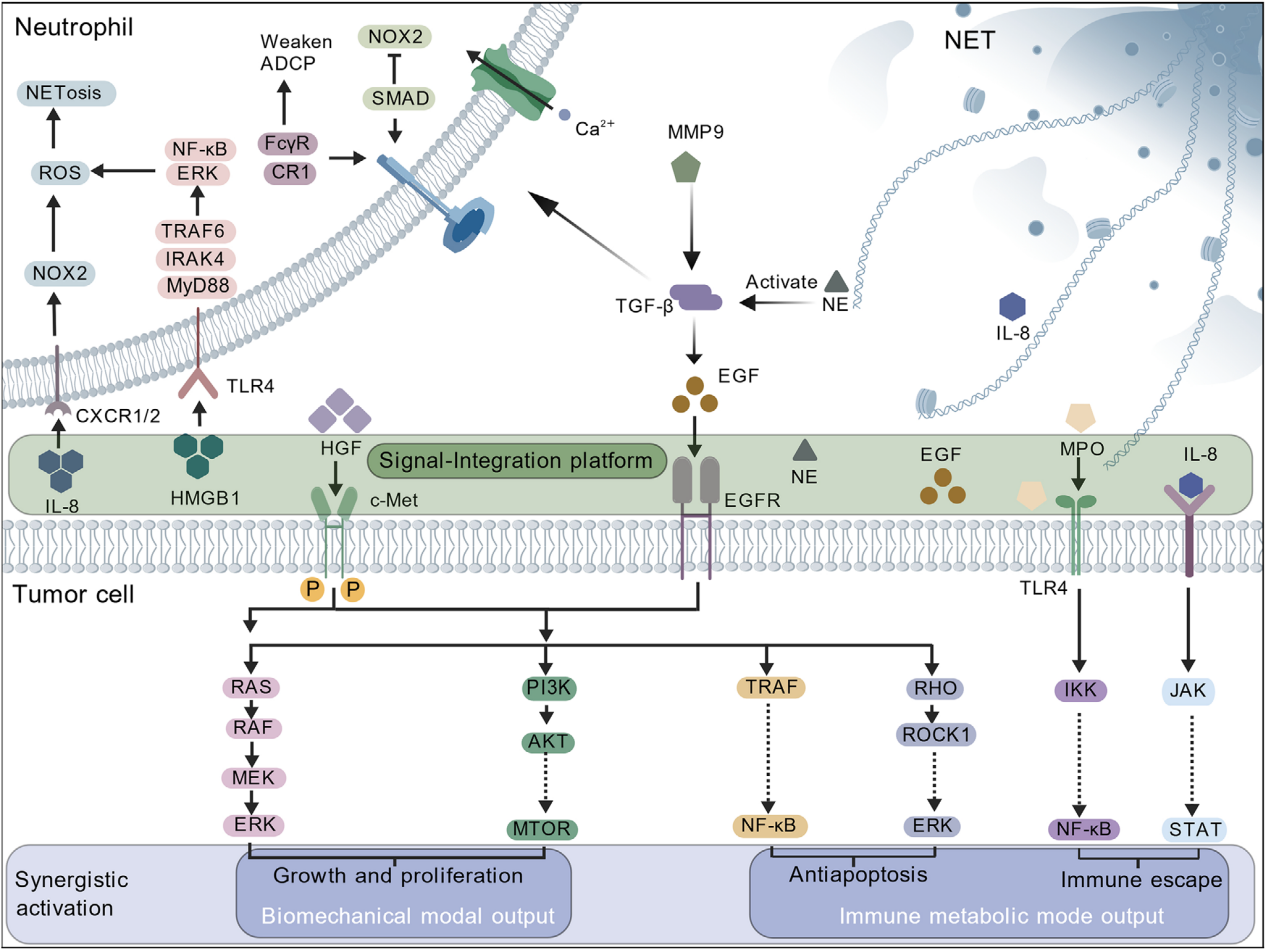
NETs as a multimodal network orchestrating tumor progression through three interconnected functional modules. The left panel depicts the immunometabolic hub where NET components including NE and HMGB1 activate TLR signaling to drive NF‐κB and HIF‐1α, promoting aerobic glycolysis and lactate accumulation. This acidotic microenvironment suppresses cytotoxic lymphocytes and regulatory T cells while promoting M2 macrophage polarization and VEGF‐driven angiogenesis, with a hypoxia–NETosis feedback loop sustaining this immunosuppressive state. The middle panel illustrates the biomechanical scaffold where integrins αvβ3 and α5β1 engage NET adhesion ligands to trigger FAK phosphorylation, activating Rho/ROCK for cytoskeletal remodeling, MAPK for proliferation, and PI3K–Akt for survival. This scaffold physically excludes T cells and NK cells while recruiting MDSCs and regulatory T cells, and NET‐associated NE and MMP‐9 degrade the extracellular matrix to facilitate tumor cell migration while promoting endothelial injury and platelet aggregation leading to a hypercoagulable state. The right panel shows the signal‐integration platform where diverse mediators including IL‐8, NE, HMGB1, MMP‐9, and TGF‐β are spatially concentrated on the DNA scaffold, enabling synergistic activation of EGFR and c‐Met to converge on PI3K–Akt and Ras–MAPK pathways, while concurrently inducing epithelial–mesenchymal transition through β‐catenin and Snail/Slug signaling to promote tumor cell migration. The bottom green panel illustrates the synergistic culmination of these modalities, where the integrated biomechanical disruption, metabolic suppression, and signaling convergence reactivate dormant cancer cells through NE‐mediated laminin cleavage, Smad4 degradation, and HMGB1 and IL‐8‐driven cell cycle re‐entry, representing the ultimate cooperative output of the multimodal network.

#### 5.3.3 Protease Resistance and Sustained Signaling

When proteases bind to NET‐DNA, they become resistant to endogenous inhibitors: NE attached to DNA resists α1‐antitrypsin, prolonging its activity in the TME, and CG similarly evades inhibition when associated with NET‐DNA [185]. This sustained proteolytic activity has multiple consequences: ongoing ECM degradation facilitates continuous invasion; sustained cleavage of immune receptors such as CD8 and NKp46 ensures prolonged immune suppression; and the extended release of matrix‐bound growth factors provides chronic stimulation for cell proliferation [186]. In tumors, neutrophil‐derived DNA enhances CG's hydrolytic activity by decreasing local protease inhibitors, and CG can activate platelets, increasing their deposition on tumor cell surfaces [187].

#### 5.3.4 Mechano‐Chemical Signal Integration

Beyond ECM degradation, NETs directly trigger EMT, a critical step in tumor aggressiveness. Laboratory experiments demonstrate that NE causes cells to lose cell‐to‐cell connections, adopt spindle‐like morphology, and activate the Wnt/β‐catenin pathway [188]. At the molecular level, NE cleaves and downregulates E‐cadherin, allowing β‐catenin to translocate to the nucleus and activate TCF/LEF transcription factors. Disruption of NETs through DNase treatment mitigates these changes, indicating that intact NETs are necessary for full EMT induction. Concurrently, the NET‐shaped microenvironment activates EMT transcription factors such as Snail, Slug, ZEB1, and Twist, which suppress E‐cadherin expression while upregulating N‐cadherin and vimentin, facilitating cytoskeletal rearrangement and enhanced cell motility. Neutrophil‐derived IL‐17 in inflamed regions promotes tumor migration and EMT via the STAT3 pathway, particularly in GC, and NET components like HMGB1 can activate NF‐κB, integrating with Wnt and TGF‐β signaling to stabilize an invasive cellular state, serves a specific function in the NET context by integrating with Wnt and TGF‐β signaling to stabilize an invasive cellular state and prevent reversion to an epithelial phenotype. Thus, NETs both initiate and sustain EMT in malignant epithelia (Figure 4). NET‐induced EMT exemplifies multimodal synergy: the biomechanical scaffold cleaves E‐cadherin via NE, the signal‐integration platform activates NF‐κB and STAT3 pathways through HMGB1 and IL‐17, and the immunometabolic hub stabilizes EMT transcription factors through lactate and hypoxia. This tripartite cooperation locks cells into a sustained invasive program, representing a key functional output of NET‐driven malignant progression.

While the primary focus here is on cancer, similar NET‐mediated tissue remodeling and barrier disruption have been documented in inflammatory bowel disease and asthma, suggesting that targeting NET‐induced structural changes may provide therapeutic benefits beyond oncology.

#### 5.3.5 Awakening Dormant Cells through Integrated Signals

Dormant tumor cells reside in specialized niches where growth‐inhibitory signals, including TGF‐β and immune surveillance mechanisms, maintain their quiescent state. In the broader context of metastasis, the failure of immune surveillance—particularly impaired activity of NK cells and CD8^+^ T cells—has been recognized as a critical enabler of metastatic outgrowth. However, the mechanical and stromal cues that complement immune escape in awakening dormant cells remain less well understood. NETs provide a unique solution by serving as a multimodal trigger that simultaneously disrupts the physical niche, disables growth‐inhibitory signaling, and recruits proinflammatory mediators. This coordinated action positions NETs as integrators that converge multiple metastasis‐promoting pathways into a single awakening signal.

The signal‐integrating capacity of NETs is particularly evident in their role in reactivating dormant tumor cells. Dormant cells, located in specialized niches where growth‐inhibitory signals maintain quiescence, can be disrupted by NETs through integrated biomechanical, metabolic, and inflammatory inputs. NE‐mediated cleavage of laminin in the perivascular niche releases dormant cells from adhesive constraints, while MMP‐9‐mediated ECM remodeling liberates proliferative signals. Concurrently, HMGB1 and IL‐8, both well‐characterized inflammatory mediators, are concentrated on the NET scaffold at levels far exceeding those in the bulk tumor interstitium, acting as potent triggers that drive dormant tumor cell cycle re‐entry [189]. This multimodal assault on dormancy—simultaneously disrupting the niche, disabling growth‐inhibitory signaling, and providing proliferative cues—explains why NETs are such potent inducers of metastatic relapse.

After the formation of a premetastatic niche, the revival of dormant tumor cells located in remote organs constitutes another significant aspect of metastatic relapse. Dormant cancer cells represent a latent reservoir of malignancy that can persist for years in a quiescent state, evading immune surveillance and therapeutic eradication [190, 191]. These cells have been identified in various solid tumors, including breast, lung, and renal cancers [191, 192, 193]. Recent findings indicate that NETs serve as powerful microenvironmental stimuli capable of disrupting the dormant state and facilitating metastatic resurgence.

Biochemical and structural processes mediated by neutrophils and NETs activate these inactive cells [194, 195]. Biochemically, proinflammatory cytokines (e.g., IL‐6, IL‐8, TNF‐α) and chemokines (e.g., CXCL1, CXCL2, CXCL5, CXCL12) secreted by activated neutrophils trigger important signaling pathways, including NF‐κB and STAT3, leading to the reentry of previously inactive cells into the cell cycle [196, 197]. Structurally, the ECM is remodeled by NET‐associated proteases, which liberate latent growth factors like VEGF and TGF‐β and expose migration‐permissive signals that support dormancy escape [198].

Among these proteolytic actions, laminin remodeling plays a critical role in the disruption of dormancy facilitated by NETs. NE and MMP‐9 cleave Laminin‐411 and Laminin‐511 in the perivascular niche, stimulating integrin α3β1 signaling, which promotes the expansion of dormant cells [199]. This targeted proteolysis is essential for reactivating the quiescent state without disrupting the network scaffolds, highlighting that both proteolysis and the physical scaffold are necessary for facilitating cytokine‐based chemotaxis. Notably, antibodies targeting laminin degradation have been found to inhibit NET‐induced awakening, emphasizing the mechanistic specificity of this process.

Beyond ECM remodeling, direct physical interactions between NETs and dormant tumor cells enhance the awakening effect. NET constituents, including histones and nonhistone proteins, interact with tumor cell surface integrins, facilitating adhesion and intracellular signaling [200]. Variations in vascular density and ECM composition across tissues influence NET accumulation and the effectiveness of contacts with dormant cells. NE serves as a core factor by cleaving laminin in the basement membrane and ECM, thereby dismantling the structural defenses of the dormancy niche [23]. Notably, NE can penetrate dormant cells, translocate to the nucleus, and degrade Smad4, a major effector of the TGF‐β pathway essential for maintaining the dormant state [201]. The loss of Smad4 inhibits TGF‐β‐mediated growth arrest, allowing tumor cells to re‐enter the proliferative cycle [202]. Enhanced proawakening effects may also arise from MMP‐9‐mediated remodeling in highly fibrotic organs, such as the liver. Furthermore, NET‐associated molecules like HMGB1 and IL‐8 contribute to this transition by binding to their respective receptors on tumor cells, delivering strong inflammatory and proliferative signals that facilitate the shift from dormancy to active growth [203, 204].

Collectively, the roles of NETs in escaping dormancy exhibit tissue specificity and dependence on various components. NETs orchestrate a progression in which the NE meshwork enables local retention and breakdown of laminin, directly targeting the gatekeepers of nuclear dormancy, including Smad4. Concurrently, MMP‐9 acts to disrupt the ECM and liberate proliferative signals. This mechanistic cascade elucidates the heightened prevalence of NET‐induced metastatic relapse in organs rich in laminin, such as the bone marrow and lungs. Consequently, targeting NETosis or its associated proteolytic machinery presents a promising therapeutic strategy to prevent metastatic reactivation in cancer patients.

The three modalities outlined—biomechanical scaffolding, immunometabolic reprogramming, and signal integration—do not function as independent entities. Instead, they are dynamically interconnected, forming a systems‐level network whose emergent properties elucidate the context‐dependent duality of NET functions, as described in Section 5.4.

### 5.4 Integration of Modalities

The three modalities—biomechanical, immunometabolic, and signal‐integrating—are dynamically interconnected through core regulatory nodes that convert inputs across these modalities into coherent functional outputs. This network architecture generates emergent properties such as signal amplification, context‐dependent outputs, and robustness.

HIF‐1α functions as a master integrator, linking the biomechanical and immunometabolic modalities. Hypoxia stabilizes HIF‐1α, driving glycolytic reprogramming to sustain ATP production necessary for NETosis while simultaneously enhancing the transcription of NE and MPO, which facilitates ECM degradation and metabolic suppression through arginine depletion and lactate generation [205]. This forms a self‐amplifying mechano–metabolic axis.

The integrin–FAK axis connects the biomechanical scaffold and the signaling hub. NET‐bound adhesion ligands initiate FAK phosphorylation, promoting cytoskeletal remodeling while scaffolding Grb2–SOS and PI3K to activate MAPK and Akt signaling, thereby amplifying growth factor signals from EGFR and c‐Met concentrated on NETs [206]. This directly couples mechanical cues to proliferative signaling.

Bidirectional crosstalk occurs through effector molecules: NE and MPO contribute to both ECM degradation and immunometabolic suppression [207]; lactate generated from glycolysis modifies NET components via histone lactylation, altering their immunostimulatory properties [208]; and HMGB1 activates TLR4 to drive both glycolytic reprogramming and synergistic signaling [209].

These interconnections facilitate signal amplification through self‐reinforcing cycles, generate context‐dependent outputs based on input combinations, and provide robustness against single‐target inhibition. Effective therapeutic intervention requires disrupting network hubs such as HIF‐1α and the integrin–FAK axis rather than targeting any single modality in isolation, thereby informing the combination strategies discussed in subsequent sections.

## 6 The Inhibitory Effect of NETs on Tumors

The dual nature of NETs—capable of both promoting and suppressing disease—is especially evident in cancer, where structures that drive metastasis and immune evasion can, under certain conditions, exert tumor‐restraining effects [210]. However, this duality extends beyond oncology. In autoimmune diseases, NETs exacerbate inflammation while potentially limiting tissue damage through the degradation of cytokines and chemokines [211, 212]. In thrombosis, NETs promote clot formation but also play a role in clot resolution and tissue repair [213]. Therefore, understanding the contextual determinants of NET function—termed “switch mechanisms”—is a central challenge that transcends individual disease boundaries. This section examines the conditions under which NETs suppress tumor growth, offering insights that may enhance our understanding of their protective roles in other pathological contexts.

### 6.1 Antitumor Mechanisms of NETs

Although neutrophils frequently contribute to tumor progression, they can also exert antitumor effects under specific conditions. As central components of innate immunity, neutrophils directly kill tumor cells and enhance adaptive immune responses through interactions with other immune cells [214]. Neutrophil‐derived NETs play a role in multiple antitumor mechanisms.

MPO, a key enzyme for NET formation, can directly induce cytotoxicity in melanoma cells and suppress tumor growth. Patients with CGD, who have defects in NADPH oxidase and consequently exhibit reduced superoxide and hydrogen peroxide production, experience increased cancer incidence [215, 216]. This highlights a functional link between MPO‐dependent oxidative pathways and the tumor‐inhibitory potential of NETs. Histones—particularly H2A, H2B, and H3—constitute the most abundant protein fraction of NETs [217]. Once released extracellularly, they can disrupt epithelial and vascular integrity and exert direct membrane‐damaging or apoptotic effects on tumor cells. The mesh‐like structure of NETs can physically trap CTCs, limiting metastatic spread [218]. DCs can capture NET‐associated tumor antigens and present them to T cells, potentially amplifying adaptive immune responses. Additionally, chemokines and damage‐associated molecular patterns (DAMPs) released from NETs promote the recruitment of monocytes, NK cells, and other effector cells, augmenting local immune activation [219].

### 6.2 Influence Of Tumor Immune Status on NET Antitumor Activity

The opposing roles of NETs—as protumorigenic versus antitumorigenic—can be reconciled by examining how different cytokine environments reshape the interconnected modalities of the multimodal framework. Under an IFN‐γ‐rich microenvironment, neutrophils polarize toward an N1‐like phenotype [220]. Within the signal‐integration platform, NETs become enriched with immunostimulatory molecules such as CXCL10, ICAM‐1, and MHC Class I antigens, facilitating DC maturation and T cell activation [136]. The immunometabolic hub shifts away from glycolytic reprogramming; instead, NETs produce less lactate and ROS, preserving T cell and NK cell function while supporting oxidative metabolism. The biomechanical scaffold does not form dense perivascular barriers that exclude lymphocytes; rather, NETs present a loose, permissive structure that allows immune cell infiltration and physical contact with tumor cells. Consequently, all three modalities converge to produce an antitumor output: enhanced immune surveillance, direct tumor cell killing via MPO‐derived hypochlorous acid, and suppression of metastatic seeding [136]. In contrast, under a TGF‐β‐dominated microenvironment, neutrophils adopt an N2‐like phenotype [221]. The signal‐integration platform now concentrates immunosuppressive cytokines such as IL‐10 and TGF‐β, along with PD‐L1, promoting Treg expansion and CD8+ T cell exhaustion [222]. The immunometabolic hub engages full glycolytic reprogramming, leading to lactate accumulation, arginine depletion, and HIF‐1α‐stabilized hypoxia, which collectively suppress effector immune cells and polarize macrophages toward M2 [223]. The biomechanical scaffold becomes dense and rigid, forming perivascular NET deposits that physically exclude cytotoxic T cells and NK cells while providing migration tracks for tumor cells [224]. In this context, these three modalities synergize to generate a protumorigenic output: immune evasion, angiogenesis, matrix remodeling, and metastatic dissemination. Therefore, the context‐dependent duality of NETs is not a paradox but a predictable consequence of how IFN‐γ and TGF‐β rewire the multimodal network. IFN‐γ drives the three modalities toward an antitumor configuration, while TGF‐β locks them into a self‐amplifying protumorigenic state. This unified explanation, grounded in the multimodal framework, resolves the long‐standing question of why NETs can both promote and hinder host health.

### 6.3 Impact Of Molecular Subtypes on NET‐Mediated Tumor Suppression

Emerging evidence indicates that NET heterogeneity—in both composition and functional consequences—extends across and within cancer types, with specific molecular subtypes exhibiting distinct NET profiles that correlate with differential clinical outcomes [111, 225]. Building on the heterogeneity data presented in Section 2, the influence of distinct NET‐associated gene signatures on the balance between tumor suppression and promotion is highlighted. In GC, studies have identified two NET‐related subtypes with divergent clinical trajectories. One subtype, characterized by high expression of C5AR1 and associated genes, correlates with poor prognosis and an immunosuppressive TME, suggesting that NETs in this context are predominantly protumorigenic. Conversely, the alternative subtype may contain NETs with antitumor potential, although this requires further functional validation [51]. Similarly, in pancreatic cancer, distinct NET subtypes characterized by PDE4B^+^, IL1B^+^, and TLR2^+^ expressions may represent functionally diverse NET populations with varying capacities for tumor suppression versus promotion [54, 226]. These findings emphasize that bulk NET quantification is insufficient; therefore, molecular subtyping of NETs—beyond simple measurement of NET burden—will be essential for predicting whether NETs in an individual patient are likely to act as allies or adversaries. In lung adenocarcinoma, distinct NET‐related gene expression patterns may influence the immune context and, consequently, the functional outcome of NET formation. The interplay between NET molecular profiles and the tumor immune microenvironment likely dictates the net effect of NETs on tumor progression [227, 228]. Future research integrating single‐cell and spatial transcriptomics with functional assays will be critical in elucidating these relationships.

### 6.4 Unanswered Questions and Therapeutic Implications

The key unanswered question is not whether NETs can exhibit antitumor effects—they clearly can—but rather which microenvironmental and molecular cues determine the predominance of one functional state over another [229]. Identifying these “switch mechanisms” could uncover strategies to favor NET‐mediated tumor suppression instead of progression. Candidates for regulation include the cytokine milieu (IFN‐γ vs. TGF‐β) [230], metabolic factors (hypoxia, lactate) [231], stromal interactions (with CAFs and TAMs) [232], and intrinsic neutrophil heterogeneity. Understanding how these factors integrate to shape NET functional polarity represents a critical research priority. Importantly, recognizing that NETs can exert antitumor effects under certain conditions has therapeutic implications. Instead of pursuing blanket inhibition of NETosis, future strategies might focus on selectively promoting the formation of antitumor NETs while suppressing protumorigenic variants. This could be accomplished through cytokine‐based therapies or by targeting pathways that drive N2 polarization [220, 221]. However, such approaches remain speculative and necessitate rigorous preclinical validation. In summary, although protumorigenic functions of NETs prevail in most advanced cancers, the antitumor potential of NETs represents an underappreciated aspect that may be harnessed therapeutically under optimal conditions [233]. A deeper understanding of the immune status and molecular subtypes governing NET functional polarity is essential for realizing this potential and for avoiding unintended consequences of NET‐targeted interventions. The multimodal framework proposed in Section 6.2 provides a mechanistic basis for understanding how cytokine contexts dictate NET polarity. Thus, future therapeutic strategies could aim to pharmacologically shift the network from a protumorigenic to an antitumorigenic configuration, for example, by blocking TGF‐β signaling or delivering IFN‐γ.

### 6.5 Critical Perspectives on NET‐Mediated Tumor Suppression

Despite the promising antitumor activities described, several critical caveats must be acknowledged. The tumor‐suppressive functions of NETs exhibit marked context dependency, varying across tumor types, experimental models, and disease stages [207]. Direct cytotoxic effects of MPO and histones have primarily been documented in melanoma models, while evidence in pancreatic cancer, colorectal cancer, and other common solid tumors remains sparse [20, 234]. It remains largely unknown whether NETs directly kill tumor cells in these malignancies or if their antitumor effects are mediated indirectly through immune cell recruitment [136]. Discrepancies among published studies further highlight the influence of experimental variables. Some studies indicate that NETs directly kill tumor cells via MPO‐derived hypochlorous acid, whereas others find that NETs primarily act by recruiting NK cells or presenting tumor antigens to DCs [235]. These differences may stem from variations in neutrophil sources, NET induction methods, tumor cell lines used, or the timing of NET addition relative to tumor challenges [236]. Furthermore, NETs generated under different stimuli can have distinct compositions—some enriched in proteases, others in histones or HMGB1—which dictate their functional output. Another important consideration is the clinical relevance of NET‐mediated tumor suppression. Most evidence comes from mouse models using transplanted tumor cell lines, which may not accurately reflect human disease. Few human studies correlate NET markers with favorable prognosis, often confounded by the dual role of NETs [237]. For instance, while a high NET burden generally predicts poor outcomes, in certain early‐stage cancers or specific treatment contexts, elevated NETs have been associated with longer survival [238]. These apparently paradoxical observations underscore that NET burden alone is insufficient as a biomarker; rather, the functional polarity of NETs, determined by the cytokine milieu and immune context, dictates their prognostic significance. Prospective clinical trials that measure NET functional polarity alongside patient outcomes are urgently needed to validate these findings.

In summary, while NETs exhibit genuine antitumor potential, this activity is highly context dependent, restricted to specific tumor types and stages, and subject to substantial variability across experimental systems. Generalizing these findings without rigorous validation in human‐relevant models could lead to misguided therapeutic strategies. Future research should focus on standardizing NET induction methods, comparing antitumor efficacy across multiple tumor types, and developing biomarkers that distinguish protective NETs from pathogenic variants.

## 7 From NET‐Targeted Agents to Precision Oncology

The preceding sections have established NETs as critical drivers of tumor progression and therapy resistance, providing a compelling rationale for therapeutic targeting. Based on this conceptual framework, current therapeutic strategies can be reclassified by the modality they primarily target. The biomechanical scaffold can be dismantled using DNase I, which degrades the DNA backbone of NETs, or FAK inhibitors, which block mechanotransductive signaling [239]. This therapeutic strategy, originally developed for cystic fibrosis where NETs contribute to airway obstruction, has been repurposed for oncology based on the recognition that NET degradation can disrupt the biomechanical scaffold modality and prevent premetastatic niche formation. The immunometabolic hub can be inhibited by PAD4 inhibitors to prevent new NET formation, MCT4 blockers to relieve lactate‐mediated T cell suppression, or arginase inhibitors to restore l‐arginine availability [240, 241]. The signal‐integration platform can be disrupted by blocking key signaling molecules such as TGF‐β, IL‐8, or HMGB1, thereby preventing synergistic receptor activation [242]. This modality‐based classification directly informs the design of rational combination strategies (Figure 6).

**FIGURE 6:**
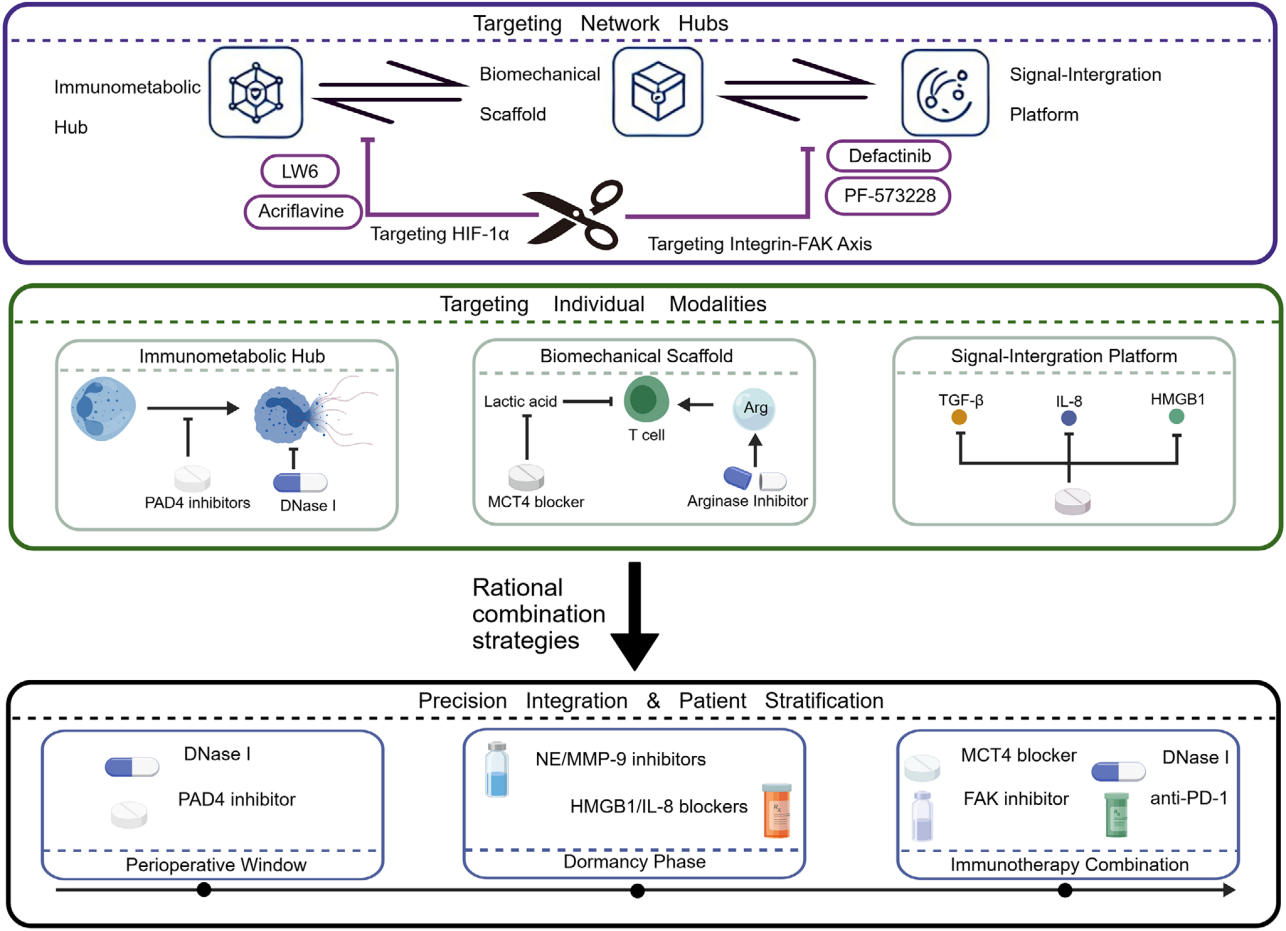
Multimodal precision intervention strategies for targeting NETs. This schematic translates the systems‐level understanding of NETs into actionable therapeutic strategies organized hierarchically. The top layer depicts the targeting of network hubs where a pair of scissors symbolizes the disruption of intermodal crosstalk achieved by inhibiting HIF‐1α with LW6 and Acriflavine and by blocking the integrin–FAK axis with defactinib and PF‐573228. The middle layer illustrates the specific targeting of individual modalities, where DNase I and PAD4 inhibitors dismantle the biomechanical scaffold, MCT4 blockers and arginase inhibitors relieve the immunometabolic hub, and neutralizing antibodies against TGF‐β, IL‐8, and HMGB1 disrupt the signal‐integration platform. The bottom layer integrates these targeted modalities into rational combination strategies adapted to distinct clinical phases, including the perioperative window to prevent acute formation of premetastatic niches, the dormancy phase to suppress dormant tumor cell reactivation, and immunotherapy combinations to reverse T cell exclusion and immune evasion. The entire framework is guided by a biomarker‐driven stratification algorithm that distinguishes patients with high NET burden and protumorigenic polarity as optimal candidates for these therapies while identifying those with protective antitumorigenic signatures who should avoid NET‐directed interventions.

### 7.1 Current and Emerging NET‐Targeting Strategies

Several classes of NET‐targeting agents are currently under preclinical and clinical investigation. PAD4 inhibitors, such as GSK484 and JBI‐589, block histone citrullination and prevent NET formation, demonstrating efficacy in models of breast cancer lung metastasis and other contexts [88, 243]. However, their clinical utility is restricted by stimulus‐specific efficacy, as PAD4‐independent NETosis pathways can arise under certain conditions. DNase‐based approaches degrade the DNA backbone of existing NETs; systemically administered DNase I has shown antimetastatic effects in preclinical models, while emerging nanoparticle‐based delivery systems aim to achieve localized NET degradation with reduced systemic exposure. These targeted delivery approaches, adapting principles from cancer nanomedicine, address a key limitation of systemic NET inhibition—the risk of compromising antimicrobial immunity at distant sites—by concentrating DNase or PAD4 inhibitors specifically within the TME. Protease inhibitors targeting NE and MMP‐9, such as sivelestat and selective MMP‐9 inhibitors, block NET‐associated enzymatic activity and ECM remodeling, with sivelestat demonstrating benefits in reducing postoperative complications and enhancing trastuzumab efficacy [244, 245]. Anti‐inflammatory modulation targeting upstream inducers of NETosis, including IL‐8/CXCR2 blockade and complement inhibition, aims to decrease NET formation at its source [233].

Despite these promising strategies, several translational challenges currently limit clinical implementation. First, the dual role of NETs in host defense raises safety concerns; systemic NET inhibition may compromise antimicrobial immunity, as evidenced by recurrent infections in patients with CGD who lack functional NADPH oxidase [70]. Second, the absence of validated biomarkers to distinguish protumorigenic from protective NETs hampers patient selection and response monitoring; circulating markers such as MPO–DNA and citrullinated histone H3 measure total NET burden but do not indicate functional polarity [138]. Third, NET heterogeneity across and within tumor types suggests that a one‐size‐fits‐all targeting strategy may prove ineffective; different tumor types exhibit distinct NET gene expression profiles and subtype compositions [51, 53, 54]. Fourth, species differences between mouse models and human tumors complicate translation; murine neutrophils produce NETs with different protease profiles and lower PAD4 dependence compared with human neutrophils, indicating that agents effective in mice may have limited efficacy in humans [246, 247]. These challenges underscore the necessity for biomarker‐driven patient stratification, localized delivery strategies, and meticulous safety monitoring in clinical trial design. MPO–DNA and citrullinated histone H3 have shown promise as disease activity markers in sepsis and SLE, suggesting that these biomarker panels could be repurposed across disease contexts, provided that functional polarity is interpreted with care.

### 7.2 Biomarkers for Patient Stratification

Precision medicine for NET‐targeted therapies necessitates validated biomarkers that quantify NET burden, distinguish protumorigenic from protective NETs, and predict therapeutic response. Circulating markers such as MPO–DNA complexes and citrullinated histone H3 are widely studied for assessing NET burden. Elevated MPO–DNA correlates with poor prognosis and resistance to immune checkpoint inhibitors (ICIs) in multiple cancers, while citrullinated histone H3 reflects PAD4‐dependent NETosis and may identify patients likely to respond to PAD4 inhibitors [188]. However, these markers measure total NET load without indicating functional polarity. Therefore, a combined stratification strategy that integrates burden markers with functional markers is proposed. NET‐associated gene signatures can effectively discriminate functional states: high C5AR1 expression identifies protumorigenic NETs associated with immunosuppression, whereas an IFN‐γ‐related signature, including CXCL10 and IFITM1, indicates antitumorigenic NETs [51]. Based on this, patients exhibiting high NET burden along with a protumorigenic signature—such as elevated MPO–DNA and high C5AR1 expression—represent optimal candidates for NET‐targeted agents, particularly when combined with immunotherapy [237]. In contrast, patients with high NET burden yet an antitumorigenic signature, such as high MPO–DNA coupled with a strong IFN‐γ‐related gene profile, may experience negation of protective NET effects; therefore, in patients whose NETs exhibit an antitumorigenic molecular signature, NET‐targeted therapies should be approached with caution, if not avoided altogether, as therapeutic disruption of protective NETs may paradoxically accelerate tumor progression. Patients showing low NET burden, as indicated by low MPO–DNA levels, are unlikely to benefit from NET‐directed interventions and should not be recommended for such treatments.

Clinical implementation faces several limitations: MPO–DNA lacks specificity since cell‐free DNA can also originate from apoptosis, and functional gene signatures necessitate prospective validation [138]. To address these challenges, standardized preanalytical protocols should be advocated, alongside combination with tumor‐specific markers such as AFP for hepatocellular carcinoma or CEA for colorectal cancer. Furthermore, the development of multiplex assays that measure both burden and polarity—quantifying MPO–DNA alongside C5AR1 and IFN‐γ protein levels—would be beneficial. Prospective trials incorporating such biomarker panels are urgently needed. Importantly, MPO–DNA and citrullinated histone H3 primarily reflect the activity of the NETosis machinery, underpinning all three modalities but most directly linked to the formation of the biomechanical scaffold. In contrast, functional markers such as C5AR1 and IFN‐γ signatures indicate the polarization of the immunometabolic hub and the signal‐integration platform, respectively. Thus, a comprehensive biomarker panel should integrate burden markers with modality‐specific functional signatures. This biomarker‐driven stratification is essential, as the robustness of the multimodal network implies that targeting a single modality without understanding its crosstalk with others is unlikely to succeed.

### 7.3 When to Target NETs in the Cancer Journey

The optimal timing of NET‐targeted interventions is influenced by disease stage and tumor type, as reflected in our multimodal framework. Perioperative interventions aim to address surgery‐induced NETosis, which facilitates the formation of a premetastatic niche [248]. This intervention primarily targets the acute establishment of the biomechanical scaffold, as surgery‐induced NETs rapidly deposit in distant organs, creating physical barriers and premetastatic niches. Preclinical studies indicate that a short course of DNase I or a PAD4 inhibitor administered around the time of surgery can effectively reduce subsequent metastasis. This time‐limited intervention avoids chronic immunosuppression and can be evaluated in trials using metastasis‐free survival as an endpoint.

Metastatic relapse prevention focuses on NET‐mediated awakening of dormant tumor cells [249]. This strategy targets the signal‐integration platform because the awakening process is driven by NE‐mediated laminin cleavage and HMGB1/IL‐8 inflammatory triggers, exemplifying classic signal integration by NETs. Chronic inflammation and tissue injury gradually engage NETosis, remodeling the laminin niche and reactivating dormant cells [250]. For high‐risk patients, such as those with minimal residual disease postsurgery, intermittent NET inhibition should be implemented every 1 to 2 weeks for 3 to 6 months. This schedule aims to thwart the microenvironmental changes that trigger relapse. Timing should also be tailored to the specific tumor type. Perioperative NET inhibition is particularly important in renal cell carcinoma and lung cancer, where surgery is often curative, yet postoperative recurrence is common. In pancreatic cancer, where late metastasis and reactivation of dormant cells prevail, preventing metastatic relapse is paramount.

Combining NET‐targeted approaches with CAR‐T therapy presents additional challenges. Dense NETs hinder CAR‐T cell infiltration, and NET‐associated histones and proteases can lead to CAR‐T apoptosis [49, 251]. A crucial lesson from unsuccessful preclinical studies highlights that the limitations of PAD4 inhibitors arise from the inherent redundancy within the multimodal network. Even when PAD4‐mediated NET formation is inhibited, the biomechanical scaffold may still obstruct CAR‐T cells due to pre‐existing NETs, while the immunometabolic hub may continue to impede T cell function through lactate and arginine depletion. Consequently, single‐agent PAD4 inhibition frequently fails to yield durable CAR‐T efficacy. Effective CAR‐T combinations must adopt a multimodal disruption strategy, such as combining a PAD4 inhibitor with DNase I and an MCT4 blocker. The timing of NET degradation relative to CAR‐T administration necessitates systematic optimization. Collectively, these timing considerations indicate that effective NET‐targeted therapy must disrupt interconnections among modalities. For example, employing perioperative DNase I can dismantle the biomechanical scaffold before it establishes a premetastatic niche. Additionally, sequencing PAD4 inhibitors with ICIs prevents new NET formation and alleviates metabolic suppression [252].

### 7.4 Rational Combination Strategies Informed by the Multimodal Framework

Effective intervention necessitates a hierarchical approach: targeting central network hubs (HIF‐1α and FAK) to disrupt intermodal crosstalk, selectively inhibiting the dominant modality relevant to a specific tumor, and rationally combining these strategies across defined clinical time windows based on biomarker stratification. Guided by the multimodal framework, therapeutic strategies can be classified into three complementary categories, each designed to dismantle the network at various levels (Figure 6).

#### 7.4.1 Targeting Network Hubs

Certain molecules function as hubs where two or more modalities converge, and targeting these hubs can simultaneously interrupt multiple modalities, thereby dismantling the network's core connectivity. HIF‐1α serves as a critical juncture between the immunometabolic hub and the biomechanical scaffold. Hypoxia stabilizes HIF‐1α, driving glycolytic reprogramming within the immunometabolic hub while enhancing the production of NE and MPO, which remodel the ECM in the biomechanical scaffold [253]. Pharmacological inhibition of HIF‐1α, utilizing compounds such as LW6 or acriflavine, effectively blocks both the metabolic switch and the mechanical remodeling that support tumor cell migration and immune exclusion [114]. This dual action disrupts the energy supply for NETosis while softening the physical barrier, exemplifying how targeting a single network node can hinder modality crosstalk. The integrin–FAK axis links the biomechanical scaffold to the signal‐integration platform. NET‐bound adhesion ligands interact with integrins, triggering FAK autophosphorylation that drives cytoskeletal remodeling in the biomechanical scaffold while simultaneously scaffolding the Grb2–SOS complex to activate MAPK signaling in the signal‐integration platform [254]. FAK inhibitors, such as defactinib and PF‐573228, not only reduce tumor cell adhesion to NETs and diminish NET‐guided migration but also abrogate the synergistic amplification of growth factor signals from EGFR and c‐Met that converge on FAK. This convergence, a well‐established feature of receptor tyrosine kinase signaling networks, is particularly significant in the NET context because the spatial colocalization of EGFR and c‐Met ligands on the NET scaffold ensures simultaneous receptor engagement and maximal FAK activation, thereby amplifying mechanotransduction beyond what occurs with sequential or spatially dispersed ligand presentation. Thus, FAK inhibition dismantles both the mechanical anchorage and the proproliferative signaling provided by NETs.

#### 7.4.2 Targeting Individual Modalities

When a single modality dominates a patient's NET phenotype, an effective strategy may involve inhibiting that modality while simultaneously preventing its communication with the other two modalities. For immune‐excluded tumors characterized by a high protumorigenic NET burden, the biomechanical scaffold becomes the dominant barrier. Degrading NETs with DNase I alone proves insufficient, as the immunometabolic hub remains active, continuously suppressing T cells through lactate and arginine depletion. A more rational approach involves combining DNase I, which dismantles the scaffold, with an MCT4 blocker to relieve lactate‐mediated suppression and an arginase inhibitor to restore l‐arginine levels. This dual intervention removes the physical barrier and severs the metabolic link that would otherwise maintain immune suppression. The proposed sequence entails administering 1 to 2 weeks of DNase I alongside the MCT4 inhibitor, followed by the addition of the arginase inhibitor. Currently, no clinical‐grade MCT4 blockers are available, although AZD3965 is undergoing early‐phase trials; preclinical optimization in NET‐high models remains a pressing need.

For tumors that are metabolically suppressed with a high protumorigenic NET burden, the immunometabolic hub plays a dominant role. A PAD4 inhibitor such as GSK484 can prevent new NET formation; however, pre‐existing NETs may still function as a biomechanical scaffold [255]. In this context, combining a FAK inhibitor to block mechanotransduction from residual NETs, along with an MCT4 blocker to relieve lactate suppression, addresses both the hub and its crosstalk with the scaffold. The treatment regimen should begin with the PAD4 inhibitor for 5 to 7 days, subsequently adding the FAK inhibitor and MCT4 blocker. This combination is specifically designed to prevent the hub from reactivating the scaffold through hypoxia–HIF‐1α signaling.

#### 7.4.3 Targeting of all Three Modalities

For tumors with a high NET burden, where all three modalities are strongly engaged—such as hypoxic, stiff, and chronically inflamed tumors—targeting a single modality or even dual modalities may prove insufficient due to network redundancy. Multimodal disruption necessitates simultaneous targeting of the biomechanical scaffold, the immunometabolic hub, and the signal‐integration platform. One prototype approach is the neutrophil‐arming nanoplatform, which degrades pre‐existing NETs while inhibiting TGF‐β signaling. In combination with anti‐PD‐1 therapy, this platform achieved a 74% reduction in tumor burden in colorectal cancer models [224]. A more conventional multimodal combination could involve DNase I to degrade the biomechanical scaffold, a PAD4 inhibitor to prevent new NET formation, a FAK inhibitor to block mechanotransduction and signal integration, an MCT4 blocker to alleviate lactate‐mediated suppression, and anti‐PD‐1 to restore T cell effector function [177, 256]. Although such a five‐drug regimen might appear impractical, rational selection based on dominant network nodes in individual patients remains feasible. For instance, a patient with elevated HIF‐1α levels alongside a high NET burden may benefit from a combination of a HIF‐1α inhibitor, DNase I, and anti‐PD‐1, given that HIF‐1α operates at the intersection of metabolic and mechanical pathways.

#### 7.4.4 Future Directions

However, all multimodal combinations involve risks of infection, impaired wound healing, and off‐target inflammation; therefore, monitoring serum procalcitonin and C‐reactive protein weekly during the first month is advisable [257]. These recommendations are drawn from preclinical models, as prospective clinical data remain lacking. Adaptive Phase I/II trials that enrich for biomarkers—including MPO–DNA, citrullinated histone H3, plasma lactate, and tumor biopsies for T cell infiltration—are crucial [258]. Such trials should include safety run‐in phases to determine maximum tolerated doses for combinations. Only through rigorous multimodal clinical testing can these carefully designed strategies be effectively translated into precision oncology.

### 7.5 Lessons From Preclinical and Clinical Setbacks

A balanced translational roadmap must also recognize studies where NET‐targeted interventions did not meet expectations, as these negative results offer critical insights for future development. For example, in melanoma patients resistant to PD‐1 inhibitors, NET‐mediated T cell exclusion was identified as a major mechanism of immune evasion across multiple cancer types. Building on this recognition, recent studies have demonstrated that NETs contribute to T cell exclusion not only through biochemical immunosuppression but also through physical barrier formation, with dense perivascular NET deposits creating spatial obstacles that prevent effector lymphocyte infiltration into tumor nests. Initial efforts to reverse this resistance using DNase I alone demonstrated limited efficacy. While degrading the physical NET barrier relieved T cell exclusion, it did not address concurrent immunometabolic suppression due to lactate accumulation and arginine depletion. These metabolic mechanisms of T cell dysfunction, well characterized in solid tumors, are significantly amplified in NET‐rich regions where neutrophil‐derived lactate and NET‐associated Arg‐1 create local microenvironments with metabolite concentrations that far exceed tumor‐wide averages, resulting in focal zones of profound immunosuppression. The failure of DNase I alone is attributable to its focus solely on the biomechanical scaffold, leaving the immunometabolic hub unaffected. Consequently, lactate and arginine depletion continued to impede T cell function even after physical barriers were removed. A more effective strategy would involve combining DNase I with an MCT4 blocker to simultaneously target both modalities.

These failures impart three critical lessons for precision oncology. First, inadequate patient stratification has diluted treatment effects. Many studies enrolled unselected populations without assessing NET burden or functional polarity, resulting in mixed outcomes. Second, overlooking NET subtype heterogeneity has proven to be a significant pitfall. Protumorigenic C5AR1‐high NETs and antitumorigenic IFN‐γ‐high NETs respond differently to the same intervention; a therapy that is effective for one subtype may be ineffective or even detrimental for another. Third, inappropriate intervention timing has compromised efficacy. Delayed administration of NET inhibitors, after the metastatic niche is fully established, may overlook critical windows for intervention. Future trials must incorporate biomarker‐based stratification, selecting patients based on both NET burden and functional signatures while aligning intervention timing with the predominant phase of NET activity.

Additionally, discrepancies between mouse models and human tumors introduce an extra layer of uncertainty. Several NET‐targeted strategies that demonstrated robust antimetastatic effects in syngeneic mouse models have failed to replicate in humanized mouse models or early clinical samples [246]. The primary reason seems to be species differences in NET composition and function. Murine neutrophils generate NETs with distinct protease profiles and exhibit lower dependency on PAD4 compared with human neutrophils [247]. Consequently, a PAD4 inhibitor effective in blocking NETosis in mice may have only limited efficacy in humans. This underscores the urgent need to validate NET‐targeting agents using humanized mouse models or ex vivo human tumor platforms before advancing to clinical trials.

In summary, failed studies teach that monotherapy is seldom adequate, patient selection cannot be neglected, NET heterogeneity must be acknowledged, and human‐relevant models are essential for successful translation. Future development should prioritize multimodal combinations, biomarker‐driven enrichment, and rigorous cross‐species validation. Only by learning from these failures can the field advance toward effective NET‐targeted therapies.

### 7.6 Integrating NET‐Targeted Strategies With Immunotherapy

The greatest promise for NET‐targeted agents lies in their integration with immunotherapy, a mechanistically compelling rationale. NETs create physical barriers that impede T cell infiltration, suppress T cell function metabolically, concentrate immunosuppressive cytokines, and recruit regulatory cell populations. Each of these effects can potentially be reversed through NET‐targeted interventions. Patient selection for immunotherapy combinations should be informed by evidence of NET‐mediated immune evasion. Candidates include patients with elevated circulating NET markers who have shown progression on checkpoint inhibitor monotherapy, as well as those with known risk factors for NET‐mediated resistance, such as obesity, chronic inflammation, or recent surgery [257, 259]. Urgent prospective validation of these selection criteria is necessary. Optimal timing and sequencing of interventions require systematic investigation. Preclinical data suggest that concurrent initiation of NET inhibition alongside checkpoint blockade generally yields superior results compared with sequential approaches, although variations may exist depending on the specific agent [260]. For example, PAD4 inhibitors, which prevent new NET formation, may necessitate adequate time for target engagement prior to checkpoint blockade [252]. Conversely, DNase I, which degrades existing NETs, may be most effective when administered immediately before or concurrently with checkpoint blockade to eliminate physical barriers at the time of T cell activation [261]. Timing is critical, with concurrent administration typically outperforming sequential approaches. The rational framework outlined above offers a roadmap for developing NET‐targeted therapies. The subsequent section surveys the current translational landscape, summarizing key preclinical evidence and emerging clinical trials that are beginning to test these concepts in patients.

## 8 Translational Advances and Clinical Trials of NET‐targeted Therapies

The multifaceted roles of NETs in cancer progression and therapy resistance have generated significant interest in developing NET‐targeted therapeutic strategies. This section summarizes crucial preclinical animal studies and emerging clinical trials focused on interventions that inhibit NET formation, degrade existing NETs, or block NET‐associated effectors. Given the rapid evolution of this field, it provides a snapshot of the current translational landscape.

### 8.1 Preclinical Evidence

An increasing body of preclinical studies has validated NETs as actionable therapeutic targets across various cancer types. These investigations have employed diverse strategies, including pharmacological inhibition of NETosis, enzymatic degradation of NET scaffolds, and blockade of NET‐associated signaling pathways. Table 1 summarizes representative preclinical studies.

**TABLE 1:** Tumor treatment strategies for NETs and progress in preclinical/clinical research.

Mechanism of action	Agent	Cancer type	Mechanism	Phase	Status	Key findings	References/NCT
Inhibition of NETosis	GSK484	BCLM	−	−	−	Antimetastatic, prolonged survival	[20]
	DPI	Various	−	−		Inhibits ROS‐dependent NETosis	[262]
	IL‐8/CXCR2 blockade	Colorectal cancer	−	−	−	Suppresses NET‐driven tumor progression, enhances immunotherapy	[233]
Degradation of NETs	DNase I	Breast cancer	−	−	−	Inhibits migration/invasion/lung metastasis	[263]
	DNase I‐coated nanoparticles	Breast cancer	−	−	−	Reduces lung metastasis	[263]
	Inhalable DNase I@Au nanoparticles	Lung cancer	−	−	−	Radiosensitization, NET degradation, metastasis inhibition	[264]
Protease inhibition	Sivelestat	Esophageal cancer, breast cancer	−	−	−	Reduces postop complications, enhances trastuzumab efficacy	[244]
	MMP‐9 inhibitor	Breast cancer	−	−	−	Inhibits invasion, angiogenesis, metastasis	[245]
Multimodal targeting	TPG	CRLM	−	−	−	Blocks NETosis + remodels TME, prolongs survival	[20]
NETs degradation (DNase I)	Dornase alfa	Refractory germ cell cancer	Degrade NET DNA backbone, restore extracellular DNA homeostasis, and enhance chemotherapy sensitivity	Phase II	Not yet recruiting	No results posted yet	NCT07121322*
Complement C3/C3b inhibition	APL‐2	Epithelial ovarian cancer	Inhibit C3/C3b, reduce neutrophil recruitment	Phase II	Ongoing	No results posted yet	NCT04919629*

*Note*: Content above the dotted line refers to preclinical studies; content below refers to clinical trials. The items marked with * are classified as clinical studies, while those without any marking are classified as preclinical studies. The table summarizes all clinical studies that use targeted NETs as a means of treating cancer. For preclinical studies, several representative mechanisms are selected and listed for summary.

Abbreviations: BCLM, breast cancer lung metastasis; CRLM, colorectal cancer liver metastasis; GSK484, a PAD4 inhibitor; DPI, diphenyleneiodonium chloride; Sivelestat, a neutrophil elastase inhibitor; TPG, tFNA–Ac‐PGP@GSK484; APL‐2, pegcetacoplan.

### 8.2 Clinical Trials

Despite compelling preclinical evidence, the clinical translation of NET‐targeted therapies remains in its early stages. Key challenges include the context‐dependent duality of NET functions, the necessity for predictive biomarkers, and concerns about compromising host defense. Several clinical trials are actively investigating NET‐related interventions, primarily focused on repurposing existing agents or evaluating novel radiopharmaceuticals that leverage NET‐associated targets (Table 1). The dual nature of NETs—as protective in infections yet pathogenic in autoimmunity and cancer—presents a shared translational challenge across all therapeutic contexts, emphasizing the need for disease‐specific and context‐dependent targeting strategies.

### 8.3 Challenges And Future Directions

The clinical translation of NET‐targeted therapies is confronted by fundamental challenges, including the context‐dependent duality of NET functions, the absence of validated biomarkers for patient selection and response monitoring, and the substantial heterogeneity of NETs across tumor types [265, 266, 267]. Additionally, optimal timing and combination strategies for these agents have yet to be systematically established. These critical issues—context‐dependent NET functions, the lack of validated biomarkers, and NET heterogeneity—are not merely logistical hurdles; they stem from the complex, interconnected network architecture elaborated throughout this review. A balanced assessment of these challenges, along with recognition of conflicting evidence and knowledge gaps, is essential for translating NET‐targeted strategies into clinical practice. The following section addresses these unresolved questions and controversies.

## 9 Outstanding Questions

Despite significant advances in understanding the biological functions of NETs in cancer, several critical questions remain unresolved. A balanced appraisal of conflicting evidence and identification of knowledge gaps are necessary for translating NET‐targeted strategies into clinical practice. This section explicitly addresses areas of the field lacking consensus and aims to temper the affirmative tone prevalent in much of the literature—including parts of this review—with a critical evaluation of contradictory findings.

### 9.1 Critical Appraisal of Contradictory Findings

A balanced interpretation of NET biology necessitates confronting inconsistencies, negative results, and unresolved controversies. This section integrates discordant findings, analyzes their underlying causes, and discusses the implications for therapeutic development.

#### 9.1.1 Stimulus‐Specific and Tumor‐Type Differences in PAD4 Dependency

The requirement for PAD4 in NETosis is not absolute and varies markedly with the activating stimulus. Under LPS or PMA stimulation, NET formation is strictly PAD4 dependent; PAD4 knockout mice exhibit severely impaired NETosis in response to these stimuli [243]. In contrast, calcium ionophore can induce NETosis even without detectable histone citrullination, indicating PAD4‐independent pathways that function under specific stimulus conditions [268]. This stimulus specificity has direct implications for therapeutic targeting: a PAD4 inhibitor effective against LPS‐driven NETosis may have little effect on NETs induced by calcium ionophore or by tumor‐derived factors that activate alternative pathways. Moreover, the dominant NETosis pathway differs across tumor types. In pancreatic cancer, NETs predominantly form via PAD4‐dependent pathways, contributing to desmoplastic fibrosis and immune exclusion; thus, PAD4 inhibitors have shown preclinical efficacy in pancreatic cancer models [269]. Conversely, certain lung cancer contexts exhibit lower PAD4 dependence, with NET formation relying more on NE/MPO axes or mitochondrial ROS. These differences underscore the necessity of patient stratification based on the dominant NETosis pathway in each tumor type to rationally utilize PAD4 inhibitors.

#### 9.1.2 NET Inhibition Fails to Improve Tumor Control

A critical yet often underappreciated observation is that NET inhibition does not uniformly enhance outcomes. Emerging evidence indicates that the functional impact of NET inhibition is highly context dependent [207]. For example, in certain early‐stage tumor models or specific cytokine environments, NETs have been shown to exert antitumorigenic functions, contributing to immune surveillance rather than promoting tumor growth [255]. In such contexts, the administration of PAD4 inhibitors may not only fail to reduce tumor burden but could also negate beneficial antitumor responses. This scenario illustrates that the functional polarity of NETs—whether pro‐ or antitumorigenic—is influenced by disease stage, immune context, and the TME. The absence of reliable biomarkers to differentiate protective from pathogenic NETs risks the indiscriminate application of NET‐targeted therapies, potentially erasing advantageous antitumor responses [270].

Even in protumorigenic contexts, single‐agent NET inhibitors often yield modest or transient effects. For instance, DNase I monotherapy in melanoma patients resistant to PD‐1 inhibitors demonstrated limited efficacy, as it degraded the physical NET barrier without alleviating concurrent immunometabolic suppression stemming from lactate accumulation and arginine depletion [271]. These negative outcomes underscore three key lessons for precision oncology: first, inadequate patient stratification diminishes treatment effects; second, neglecting NET subtype heterogeneity leads to variable outcomes; and third, inappropriate intervention timing—such as administering NET inhibitors too late after the formation of the metastatic niche—misses critical windows of opportunity.

#### 9.1.3 Risks of Systemic NET Targeting

Systemic NET inhibition carries substantial risks that must be weighed against potential benefits. Patients with CGD, who are unable to generate the ROS burst necessary for classical NETosis, suffer from recurrent, life‐threatening infections [272]. While partial inhibition may differ from complete deficiency, these scenarios highlight the essential protective role of NETs. Beyond infection risks, DNase I treatment presents unanticipated hazards. In patients with inflammatory bowel disease, DNase levels rise concurrently with NETs in the colon, and DNase treatment releases NET‐related proteins, particularly MPO and histones, which synergistically damage vascular ECs in vitro. Administration of DNase in a DSS mouse model exacerbated colon injury and increased vascular permeability, effects that were inhibited by suppressing DNase activity [273]. This demonstrates that degrading NETs is not inherently advantageous, as the release of cytotoxic mediators may result in off‐target tissue injury.

Given these risks, localized targeting strategies that confine NET inhibition to the TME while preserving systemic antimicrobial functions represent a promising direction. Nanotechnology‐based platforms, such as framework nucleic acid nanoplatforms targeting neutrophils and PAD4, can orchestrate intra‐ and extra‐tumoral actions to suppress primary tumors and liver metastasis, facilitating tumor‐specific NET inhibition. Similarly, intratumoral injection of DNase I or antibody‐modified DNase I micelles that specifically recognize NETs has been developed to minimize systemic exposure. While these approaches remain in preclinical stages, they underscore the feasibility of spatially confined NET targeting. Future research should prioritize the development of tumor‐selective delivery systems, including nanoparticle‐encapsulated inhibitors and injectable hydrogels, to achieve effective NET inhibition without compromising host defense.

### 9.2 Unresolved Questions

Several fundamental questions require attention as the field progresses toward clinical translation. First, what is the true heterogeneity of NETs both across and within different cancer types? Recent single‐cell and spatial transcriptomic studies have revealed unexpected diversity in neutrophil states, yet the implications for NET structural and functional heterogeneity remain unexplored. Second, what are the kinetics of NET formation and clearance within the TME? Most studies provide static snapshots; longitudinal imaging could elucidate whether NETs are chronic deposits or dynamic structures that undergo formation‐clearance cycles. Third, is it possible to develop NET subtype‐specific biomarkers to guide patient selection? While MPO–DNA complexes and citrullinated histone H3 capture total NET burden, they do not differentiate between protumorigenic and protective NETs. Fourth, what constitutes the optimal combination strategy for NET‐targeted agents? Preclinical evidence suggests benefits when combined with ICIs, but the timing, sequence, and specific partner agents require systematic optimization. Finally, can nanotechnology‐based delivery systems achieve localized NET inhibition within tumors while preserving systemic antimicrobial functions? Addressing these questions will necessitate collaborative efforts that integrate mechanistic dissection, advanced imaging, biomarker development, and thoughtfully designed clinical trials.

In summary, the transition from understanding NET biology to therapeutic application is fraught with unresolved questions and potential pitfalls. Recognizing these limitations constitutes a strength, directing research toward the most critical gaps and guiding clinical development toward strategies most likely to succeed. The upcoming decade of NET research must prioritize mechanistic clarity, context‐dependent analysis, and rigorous translational validation over broad claims of therapeutic potential. While this review primarily focuses on cancer as a model for understanding NET biology and therapeutic targeting, the principles articulated raise questions applicable across various diseases. Do the same NET subtypes identified in pancreatic and GCs exist in autoimmune diseases, and do they carry comparable prognostic and therapeutic implications? Can biomarkers for NET burden developed in oncology—such as MPO–DNA complexes and citrullinated histone H3—be adapted to monitor disease activity in lupus or RA? Will PAD4 inhibitors, which show promise in preclinical cancer models, also yield efficacy in autoimmune diseases, and will the associated safety concerns (such as infection risk and impaired wound healing) be consistent or context dependent? Addressing these cross‐disciplinary questions will require collaborative efforts that transcend traditional disease boundaries, recognizing NETs as a shared mechanistic thread linking diverse pathological conditions.

Beyond cancer, many of the unresolved questions highlighted above extend into nontumor contexts. The same NET subtypes identified in pancreatic and GCs may also exist in autoimmune diseases such as SLE and RA, with similar prognostic and therapeutic implications [274]. Biomarkers of NET burden developed for oncology—namely, MPO–DNA complexes and citrullinated histone H3—could potentially be repurposed for monitoring disease activity in conditions like sepsis or vasculitis. PAD4 inhibitors currently being explored in cancer contexts may also demonstrate efficacy in autoimmune and thrombotic disorders [275]; however, safety concerns should be carefully re‐evaluated in each context. Addressing these cross‐disciplinary questions will require efforts that go beyond traditional disease boundaries, recognizing NETs as a unifying mechanistic element across diverse pathological conditions.

## 10 Conclusions and Perspectives

### 10.1 Positioning of the Present Review Within the Existing NET Literature

In recent years, several authoritative reviews have thoroughly documented the roles of NETs in cancer progression. Two notable examples include a review concentrating on NETs and metabolic reprogramming in renal cell carcinoma, detailing how NET‐driven metabolic alterations promote tumor growth and immune evasion specifically in kidney cancer, and another providing a comprehensive overview of NETs in health and disease, summarizing their functions across infectious, inflammatory, and malignant conditions [82, 276]. Like most existing reviews, these publications follow a traditional descriptive structure: NET formation, downstream pathogenic effects, and therapeutic targeting. However, neither has sought to fundamentally reconceptualize NETs at a systems level. The present review offers a conceptual advance that distinguishes it from previous literature. Rather than merely cataloging NET components, formation pathways, and effector functions, a unifying multimodal framework is proposed that repositions NETs as biomechanical scaffolds, immunometabolic hubs, and signal‐integration platforms. This framework does not simply reorganize existing knowledge; it introduces a novel way of understanding how NETs sense, integrate, and execute context‐dependent outputs.

Specifically, the earlier renal cell carcinoma review discussed NET‐induced metabolic reprogramming as a linear pathway, whereas the present work situates glycolysis and lactate accumulation within the immunometabolic hub, explicitly linking it to the biomechanical scaffold through hypoxia–HIF‐1α crosstalk and to the signal‐integration platform via histone lactylation. The health‐and‐disease review characterized NETosis mechanisms, such as ROS generation and PAD4 activation, as isolated molecular events. In contrast, this review reinterprets these mechanisms as gatekeepers for signal integration, illustrating how the intensity and duration of ROS and PAD4 activity dictate whether NETs adopt predominantly biomechanical, metabolic, or inflammatory phenotypes. Crucially, the treatment of core NETosis mechanisms—ROS, PAD4, and NE/MPO—does not merely reiterate earlier reviews. Instead, these mechanisms are embedded within the multimodal framework, with specific contributions to each modality discussed. For example, PAD4‐mediated histone citrullination is explained not only as a facilitator of chromatin decondensation but also as a stabilizer of the biomechanical scaffold by modifying chromatin charge density and fiber rigidity, while simultaneously participating in the immunometabolic hub through connections to histone lactylation. ROS, traditionally viewed as a signaling molecule for NETosis, is recontextualized here as a key driver of glycolytic reprogramming within the immunometabolic hub, as well as a modulator of the biomechanical scaffold's structural integrity. This integration circumvents the redundancy associated with isolated mechanism recitation, using the framework to generate novel insights.

Moreover, while prior reviews typically organized content along the conventional narrative progression from NET formation to tumor promotion to therapeutic targeting, this review structures the discussion around the conceptual framework itself. Phenomena previously treated as separate chapters—such as immune suppression, ECM remodeling, angiogenesis, and metastasis—are revealed as interconnected manifestations of the same underlying modalities. The therapeutic section also transcends a mere cataloging of inhibitors, proposing rational combination strategies grounded in the multimodal framework and addressing critical issues of patient selection, intervention timing, and biomarker development that earlier reviews have largely overlooked. In essence, this review does not reiterate known pathogenic roles of NETs; rather, it fundamentally reorients the way the field conceptualizes NET biology—as an integrated signal‐processing network, where its emergent properties clarify both its complexity and its therapeutic vulnerability. This conceptual advance, coupled with the explicit differentiation from existing authoritative reviews, positions the present work as a genuinely field‐advancing contribution rather than a redundant summary.

The multimodal framework developed within this review extends beyond cancer. In autoimmune diseases, NETs function as signaling platforms that present self‐antigens and drive interferon production. In cystic fibrosis, they create hypoxic, lactate‐rich environments that alter metabolism and damage airways. In thrombosis, NETs form physical scaffolds that trap platelets and promote clot formation. Consequently, the therapeutic strategies informed by this framework—encompassing multimodal disruption, biomarker‐driven stratification, and localized delivery—are applicable across this spectrum of diseases. By recognizing NETs as a shared mechanistic thread, future research can facilitate the translation of insights from oncology to other fields while also applying lessons from autoimmunity and inflammation to refine cancer therapies. This cross‐disciplinary perspective will be essential for realizing the full therapeutic potential of NET‐targeted interventions.

### 10.2 Main Conclusions

This review has traced the progression from the molecular building blocks of NETs to their complex mechanisms in shaping the TME, ultimately addressing emerging therapeutic strategies. The central contribution is a systems‐level framework that repositions NETs as an integrated signaling system. Rather than merely cataloging their numerous pathogenic functions, NETs are shown to operate through three interconnected roles: as biomechanical scaffolds that remodel tissue and activate mechanotransduction; as immunometabolic hubs that reprogram cellular energy use and mediate immune suppression; and as signal‐integration platforms that concentrate diverse environmental cues to create localized signaling hotspots. These roles are interconnected, forming a network whose emergent properties—signal amplification, context‐dependent output, and robustness—explain the varied and sometimes opposing effects of NETs depending on the situation. This systems‐level perspective advances the field beyond descriptive catalogs toward a more comprehensive understanding of NET functionality. Conceptualizing NETs as signal processors elucidates their historically noted but poorly explained “dual role.” NETs do not uniformly exert positive or negative influences on cancer; their effects depend on the specific interplay of physical, metabolic, and inflammatory inputs they encounter. During early, short‐term inflammation characterized by minimal tissue stiffness, NETs predominantly aid in fighting infection and supporting immune defense. However, as inflammation becomes chronic, tissue stiffness increases, and oxygen levels decrease, the network shifts toward facilitating tumor growth and metastasis [207]. This dual functionality clarifies why structures that trap bacteria may also activate dormant cancer cells and promote their growth in new locations. Understanding the “switch mechanisms” that dictate which role of NETs prevails is among the most pressing questions in the field. Resolving this could unveil new strategies to favor NET‐mediated tumor suppression over progression. Several key challenges and opportunities remain on the horizon. Improved tools are essential for studying NET heterogeneity, with single‐cell and spatial technologies beginning to unveil variations in NET subtypes across and within tumors. Linking these molecular profiles to specific functional outcomes will be crucial for predicting whether NETs in individual patients act as allies or adversaries. Clinically applicable biomarkers are also urgently needed. While MPO–DNA complexes and citrullinated histone H3 show promise, they currently measure total NET burden without distinguishing protective from harmful types [138]. Standardizing these assays and validating them in prospective trials is critical. Additionally, careful consideration of the timing and methods for targeting NETs is necessary. Opportunities presented by the perioperative window, periods of dormant cell latency, and combinations with immunotherapy each come with distinct challenges. Preclinical studies must move beyond proof‐of‐concept to systematically evaluate timing, sequence, and optimal combination strategies. Furthermore, safety concerns should be addressed proactively. NETs have evolved to protect against infection and assist in wound healing; excessive inhibition could lead to significant harm. Developing strategies to target tumor‐associated NETs while preserving protective ones—potentially through localized delivery or using agents that specifically act within the tumor environment—will be pivotal for advancing therapeutic approaches.

The ideas developed here extend beyond cancer, with the same multimodal framework applicable to understanding NETs in autoimmune diseases, chronic inflammation, and thrombosis. In lupus, NETs serve as signaling platforms that present self‐antigens and drive interferon production. In cystic fibrosis, they create hypoxic, lactate‐rich environments that alter metabolism and damage airways. In thrombosis, NETs form physical scaffolds that capture platelets and promote clot formation. The tools and strategies developed for cancer may also be applicable to these other diseases. Conversely, insights gained from studying NETs in autoimmunity and inflammation could inform novel treatment approaches for cancer. Moving forward, enhanced cross‐talk between these different areas will be beneficial. By recognizing NETs as a shared element linking diverse diseases, concerted efforts can translate mechanistic insights into tangible patient benefits. Although the path from understanding to treatment is rarely straightforward, a clearer view of how NETs function as a system enables better navigation of this journey.

In essence, NETs operate as a tripartite signaling network within the TME. As biomechanical scaffolds, they remodel tissue architecture and activate mechanotransduction; as immunometabolic hubs, they reprogram cellular metabolism and link it to immune suppression; and as signal‐integration platforms, they concentrate diverse cues to amplify oncogenic signaling. These modalities are not independent; they are dynamically interconnected through core nodes such as HIF‐1α and the integrin–FAK axis, which translate microenvironmental inputs into coherent functional outputs. This systems‐level perspective demonstrates that therapeutic efficacy relies on disrupting the interconnections that sustain the protumorigenic network state rather than merely blocking any single modality. Consequently, effective strategies must encompass multimodal targeting, guided by biomarker‐driven patient stratification and optimized intervention timing, to dismantle the network while preserving essential host defense.

## Author Contributions

H.F. and Y.T. collected and interpreted the literature and drafted the original manuscript. H.F., J.H., and B.T. critically reviewed and edited the manuscript. Y.T., B.L., and X.K. prepared the figures. All authors contributed to the conceptualization and overall structure of the review, participated in literature analysis, revised the manuscript, and approved the final version.

## Funding

This work was supported by grants from the National Natural Science Foundation of China (No. 82302332) and Yunnan Provincial Clinical Medical Research Center for Radiation and Treatment (No. 202505AJ310002).

## Ethics Statement

The authors have nothing to report.

## Conflicts of Interest

The authors declare no conflicts of interest.

## Data Availability

The authors have nothing to report.
